# Protein co-expression network-based profiles revealed from laser-microdissected cancerous cells of lung squamous-cell carcinomas

**DOI:** 10.1038/s41598-021-99695-x

**Published:** 2021-10-12

**Authors:** Toshihide Nishimura, Kiyonaga Fujii, Haruhiko Nakamura, Saeko Naruki, Hiroki Sakai, Hiroyuki Kimura, Tomoyuki Miyazawa, Masayuki Takagi, Naoki Furuya, Gyorgy Marko-Varga, Harubumi Kato, Hisashi Saji

**Affiliations:** 1 Department of Translational Medicine Informatics, St. Marianna University School of Medicine, Kawasaki, Kanagawa 216-8511 Japan; 2 Department of Chest Surgery, St. Marianna University School of Medicine, Kawasaki, Kanagawa 216-8511 Japan; 3 Laboratory of Analytical Chemistry, Daiichi University of Pharmacy, Fukuoka, Fukuoka 815-8511 Japan; 4 Department of Pathology, St. Marianna University Hospital, Kawasaki, Kanagawa 216-8511 Japan; 5 Division of Respiratory Medicine, Department of Internal Medicine, St. Marianna University School of Medicine, Kawasaki, Kanagawa 216-8511 Japan; 6 Clinical Protein Science & Imaging, Biomedical Centre, Department of Biomedical Engineering, Lund University, BMC D13, 221 84 Lund, Sweden; 7 Tokyo Medical University, Tokyo, 160-0023 Japan; 8 International University of Health and Welfare, Tokyo, 107-8402 Japan

**Keywords:** Cancer, Cell biology, Computational biology and bioinformatics, Molecular biology, Systems biology, Biomarkers, Diseases, Medical research, Molecular medicine, Oncology

## Abstract

No therapeutic targets have been identified for lung squamous cell cancer (SqCC) which is the second most prevalent lung cancer because its molecular profiles remain unclear. This study aimed to unveil disease-related protein networks by proteomic and bioinformatic assessment of laser-microdissected cancerous cells from seven SqCCs compared with eight representative lung adenocarcinomas. We identified three network modules significant to lung SqCC using weighted gene co-expression network analysis. One module was intrinsically annotated to keratinization and cell proliferation of SqCC, accompanied by hypoxia-induced aerobic glycolysis, in which key regulators were activated (*HIF1A*, *ROCK2*, *EFNA1-5*) and highly suppressed (*KMT2D*). The other two modules were significant for translational initiation, nonsense-mediated mRNA decay, inhibited cell death, and interestingly, eIF2 signaling, in which key regulators, *MYC* and *MLXIPL*, were highly activated. Another key regulator *LARP1*, the master regulator in cap-dependent translation, was highly suppressed although upregulations were observed for hub proteins including EIF3F and LARP1 targeted ribosomal proteins, among which PS25 is the key ribosomal protein in IRES-dependent translation. Our results suggest an underlying progression mechanism largely caused by switching to the cap-independent, IRES-dependent translation of mRNA subsets encoding oncogenic proteins. Our findings may help to develop therapeutic strategies to improve patient outcomes.

## Introduction

Non-small-cell carcinoma (NSCLC) is a common cause of death globally^1^. Lung squamous cell carcinoma (SqCC) accounts for approximately 30% of NSCLCs^2^ and is the second most prevalent type of lung cancer. SqCC tumors usually occur in the central part of the lung or one of the main airways, the bronchus, and are more strongly associated with smoking than any other type of NSCLC. Currently, patients with SqCC are treated with various anticancer drugs, such as molecularly targeted drugs, immune checkpoint inhibitors, cytotoxic chemotherapy, and combination therapies of these drugs. Ninety-seven percent of cases resistant to chemotherapy were attributable to smoking^3^ and, recently, next-generation sequencing has been used to easily detect some oncogenic driver mutations from small biopsy samples in clinical practice^4^. However, most cases of oncogenic-driven lung cancer are non-squamous NSCLC.

On the other hand, potentially actionable oncogenic alterations have been identified in lung SqCC such as fibroblast growth factor receptor 1 (FGFR1) amplification, MET amplification, phosphatidylinositol-4,5-bisphosphate 3-kinase, catalytic subunit alpha (PIK3CA) mutation/amplification, and discoidin domain receptor tyrosine kinase 2 (DDR2) mutation^5–7^. Although these oncogene aberrations might be useful as prognostic factors, no molecular targeted therapy has been established for SqCC. Therefore, the overall survival of SqCC is relatively shorter than that for non-squamous NSCLC. The molecular pathogenesis of SqCC has not been understood, and no targeted therapeutics are available to address acutely unmet medical needs. To improve the outcomes of SqCC patients, it is important to further understand the molecular profiles of SqCC to develop an effective therapeutic strategy.

Notable advances in high-accuracy mass spectrometry (MS) have made clinical proteomics feasible, allowing shotgun sequencing and the quantitative analysis of proteins expressed in clinical specimens. Proteome data obtained by this MS-based protein sequencing can be used to identify key disease-related proteins and therapeutic targets^8^. The main aim of this study was to identify profiles of protein co-expression networks significantly associated with lung SqCC compared to lung papillary predominant adenocarcinoma (PPA), as representative lung adenocarcinoma. We collected target cells of a certain type from sections of formalin-fixed paraffin-embedded (FFPE) cancer tissues using the laser microdissection technique (Supplementary Fig. S1), which characterized both SqCC and PPA tumors, followed by label-free spectral counting and identification-based semiquantitative shotgun proteomic analysis. Weighted gene co-expression network analysis (WGCNA)^9^, an unsupervised clustering method based on the correlation network of gene and/or protein expression, was performed to identify data-driven protein co-expression networks.

## Results and discussion

### Proteome datasets of lung SqCCs and PPAs

MS-based proteomic analysis was conducted on the FFPE tissue specimens comprising seven SqCCs and eight PPAs. These specimens were selected for their preserved condition, tumor area, and well-clarified pathological diagnosis (Table 1). Presurgical treatment was not performed for any of these lung adenocarcinomas. Statistical t-test on the smoking Brinkmann index (BI) exhibited that SqCC was significantly associated with the extent of smoking (*p* = 0.018).

**Table 1:** Clinicopathological information.

Sample no.	Age (years)	Sex	Histological type	Surgical method	Location	Tumor size on CT (mm)	Clinical TNM classification			Clinical stage	Smoking index (BI)	*EGFR* mutation status	
							c-T	c-N	c-M				
**(A) SqCC (*n* = 8)**													
SqCC_T60	76	M	SqCC	Radical lobectomy	LS6	21	cT1b	cN0	cM0	cIA	4400	Unknown	
SqCC_T62	74	M	SqCC	Radical lobectomy	LS6	20	cT1a	cN0	cM0	cIA	1000	Unknown	
SqCC_T63	56	M	SqCC	Limited resection	LS1 + 2	15	cT1a	cN0	cM0	cIA	720	Unknown	
SqCC_T64	65	M	SqCC	Radical lobectomy	RS4	19	cT1a	cN0	cM0	cIA	900	Unknown	
SqCC_T66	60	M	SqCC	Radical lobectomy	LS6	21	cT1b	cN0	cM0	cIA	800	Unknown	
SqCC_T67	63	F	SqCC	Radical lobectomy	RS3	12	cT1a	cN0	cM0	cIA	840	Unknown	
SqCC_T68	77	M	SqCC	Radical lobectomy	LS4	20	cT1a	cN0	cM0	cIA	500	Unknown	
		M (85.7%) / F (14.3%)											
Average ± SD	67.3 ± 8.4					18.3 ± 3.5							
**(B) PPA (*n* = 7)**													
PPA_T10	46	F	PPA (AD)	Radical lobectomy	LS10	26	cT1b	cN0	cM0	cIA	0	Positive	exon 21 L858R, exon20 T790M
PPA_T11	61	F	PPA (AD)	Radical lobectomy	LS9	16	cT1a	cN0	cM0	cIA	0	Positive	exon19 deletion E746-A751del
PPA_T12	71	F	PPA (AD)	Radical lobectomy	RS3	26	cT1b	cN0	cM0	cIA	0	Positive	exon19 deletion E746-A751del, S752I (TCT → ATT)
PPA_T13	73	F	PPA (AD)	Radical lobectomy	LS1 + 2 LS9	40, 22	T2a, T1b	cN0	cM0	cIB	0	Negative	
PPA_T14	63	F	PPA (AD)	Radical lobectomy	RS8	13	cT1a	cN0	cM0	cIA	0	Positive	exon 21 L858R
PPA_T15	68	F	PPA (AD)	Radical lobectomy	RS2	26	cT1b	cN0	cM0	cIA	0	Positive	L858R
PPA_T16	73	M	PPA (AD)	Radical lobectomy	RS4	10	cT1a	cN0	cM0	cIA	1060	Negative	
PPA_T17	57	F	PPA (AD)	Radical lobectomy	RS1	27	cT1b	cN0	cM0	cIA	0	Negative	
		M (12.5%)/F (87.5%)											
Average ± SD	64.0 ± 9.3					18.0 ± 9.9							
**Group comparison**													
*p*-value (t-test)	0.244					0.234					0.018		

*SqCC* squamous cell carcinoma, *PPA* papillary predominant adenocarcinoma, *AD* adenocarcinoma, *BI* Brinkmann Index.

**Figure 1:**
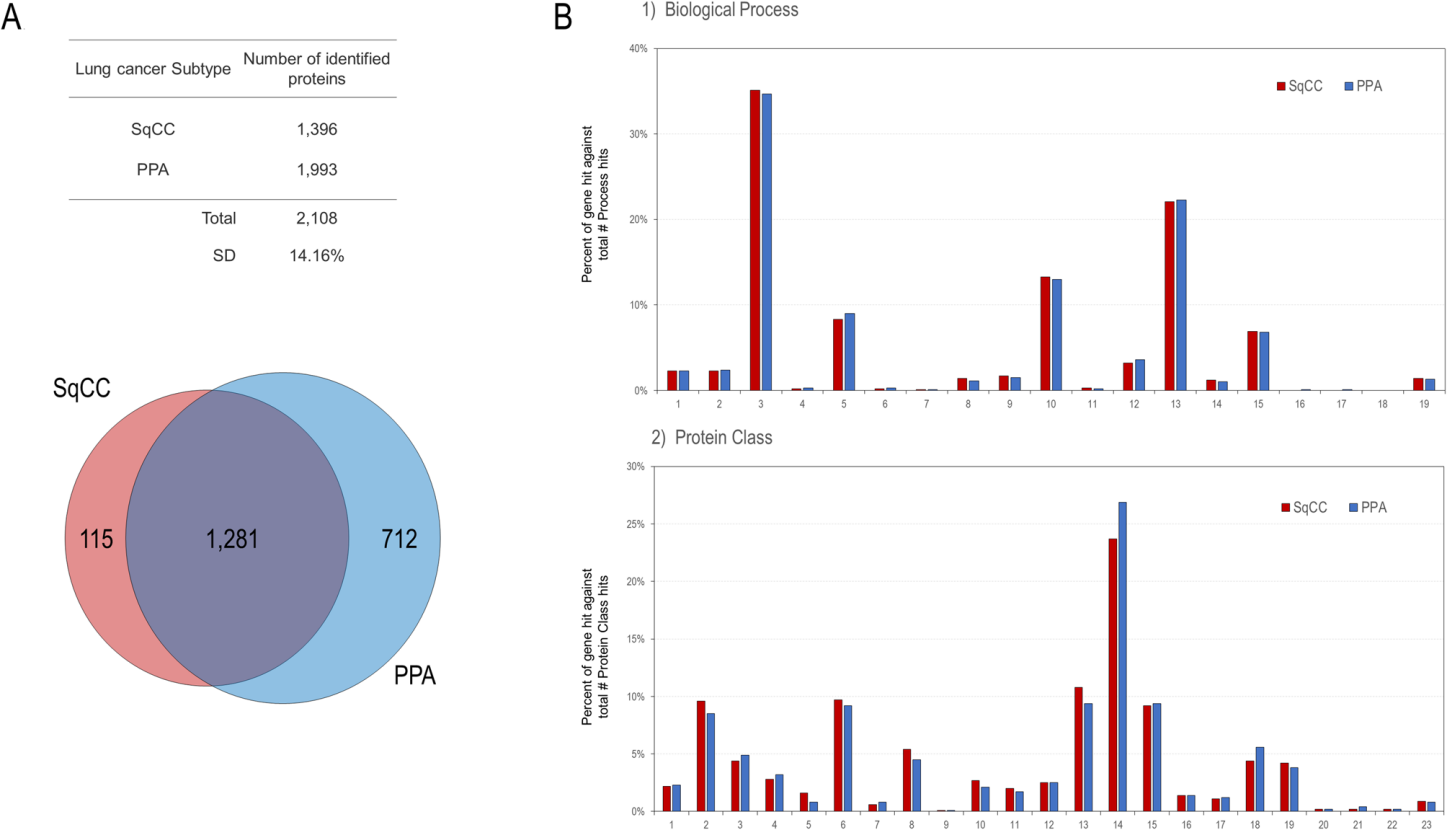
Venn map and hierarchical clustering of the identified proteins. (**A**) Venn map of the identified proteins. (**B**) Gene ontology (GO) analysis of the identified proteins for SqCC and PPA. (1) Biological process. 1, developmental process (GO:0032502); 2, multicellular organismal process (GO:0032501); 3, cellular process (GO:0009987); 4, reproduction (GO:0000003); 5, localization (GO:0051179); 6, reproductive process (GO:0022414); 7, multi-organism process (GO:0051704); 8, biological adhesion (GO:0022610); 9, immune system process (GO:0002376); 10, biological regulation (GO:0065007); 11, growth (GO:0040007); 12, signalling (GO:0023052); 13, metabolic process (GO:0008152); 14, interspecies interaction between organisms (GO:0044419); 15, response to stimulus (GO:0050896); 16, pigmentation (GO:0043473); 17, biological phase (GO:0044848); 18, behavior (GO:0007610); 19, locomotion (GO:0040011). (2) Protein class. 1, extracellular matrix protein (PC00102); 2, cytoskeletal protein (PC00085); 3, transporter (PC00227); 4, scaffold/adaptor protein (PC00226); 5, cell adhesion molecule (PC00069); 6, nucleic acid metabolism protein (PC00171); 7, intercellular signal molecule (PC00207); 8, protein-binding activity modulator (PC00095); 9, viral or transposable element protein (PC00237); 10, calcium-binding protein (PC00060); 11, gene-specific transcriptional regulator (PC00264); 12, defense/immunity protein (PC00090); 13, translational protein (PC00263); 14, metabolite interconversion enzyme (PC00262); 15, protein-modifying enzyme (PC00260); 16, chromatin/chromatin-binding, or -regulatory protein (PC00077); 17, transfer/carrier protein (PC00219); 18, membrane traffic protein (PC00150); 19, chaperone (PC00072); 20, cell junction protein (PC00070); 21, structural protein (PC00211); 22, storage protein (PC00210); 23, transmembrane signal receptor (PC00197).

A total of 2108 proteins were identified, among which 1281 (60.8%) were commonly expressed in the cancerous cells of both SqCC and PPA. One hundred and fifteen (5.5%) and 712 (33.8%) were unique to SqCC and PPA, respectively (Fig. 1A).

We subjected 1396 and 1993 proteins expressed in SqCC and PPA, respectively, to gene ontology (GO) analysis using the Protein Analysis Through Evolutionary Relationships (PANTHER) version 16.0 software program (The Thomas Lab, University of Southern California, Los Angeles, CA, USA)^10^, and the results were notably similar between the two subtypes (Fig. 1B). Common to both subtypes, proteins were abundantly associated with the cellular process, localization, biological regulation, metabolic process, and response to stimulus in the biological process (GO), and with the cytoskeletal protein, transporter, nucleic acid metabolism protein, protein-binding activity modulator, translational protein, metabolite interconversion enzyme, protein modifying enzyme, membrane traffic protein, chaperone, and hydrolase in the protein class (GO) (Fig. 1B).

### Identification of data-driven key protein network modules by WGCNA

**Figure 2:**
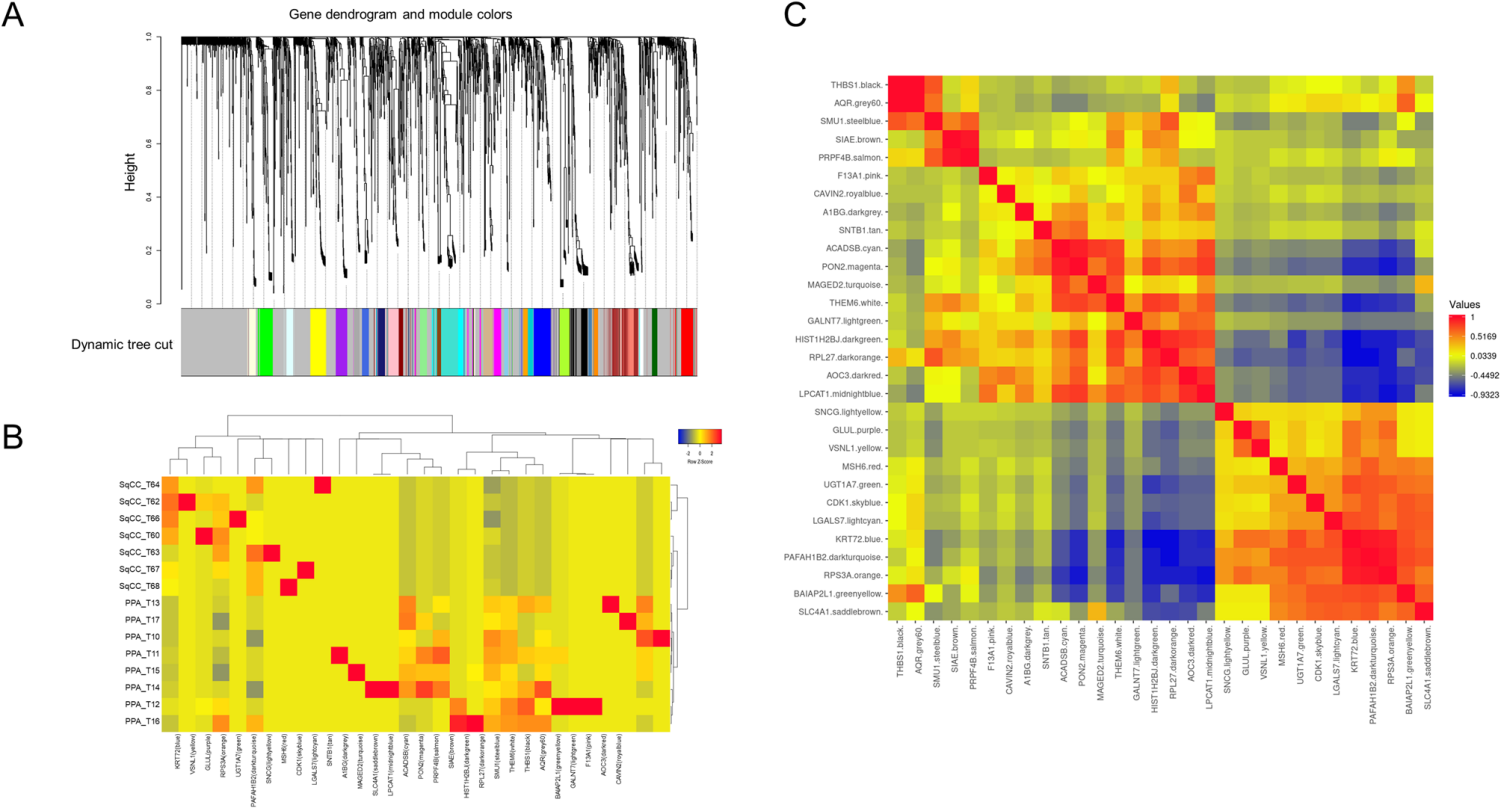
Protein network modules identified by weighted gene co-expression network analysis (WGCNA). (**A**) Protein dendrogram obtained by clustering the dissimilarity based on consensus topological overlap with the corresponding module. Colored rows correspond to the 30 modules identified. (**B**) Heatmap of semiquantitative expressions of module eigen proteins with samples. (**C**) Pairwise correlations between the modules in the heatmap of eigen proteins in module membership.

We identified 30 protein modules by constructing weighted protein co-expression networks, in which all the identified proteins were clustered (Fig. 2A). The WGCNA analysis was performed with a soft threshold power of 25 selected for approximate scale-free topology, a minimum module size of 15, and a module detection sensitivity *deepSplit* of 4. The traits used in the WGCNA analysis were the lung cancer subtypes, SqCC and PPA. Correlations were obtained between resultant modules and traits to identify the protein modules significant to respective traits. A heatmap of the eigen protein expressions and samples (Fig. 2B) and pairwise correlations between the modules regarding eigen-protein expressions (Fig. 2C) are presented respectively.

We identified nine modules that showed high and/or moderate correlations (correlation: |*r*|> 0.5) and statistical significances (multiple testing correction with the Benjamini–Hochberg method: *q*-value < 0.05) with clinical traits (Supplementary Fig. S2). We focused on the protein co-expression network modules significantly associated with lung SqCC, which were found to be WM26, WM27, and WM28 (indicated by the red dashed squares) whereas six modules, WM11, WM13, WM15, WM16, WM17, and WM18 (indicated by the blue dashed squares), were significant to PPA.

### Functional enrichment analysis of the PPI networks

**Figure 3:**
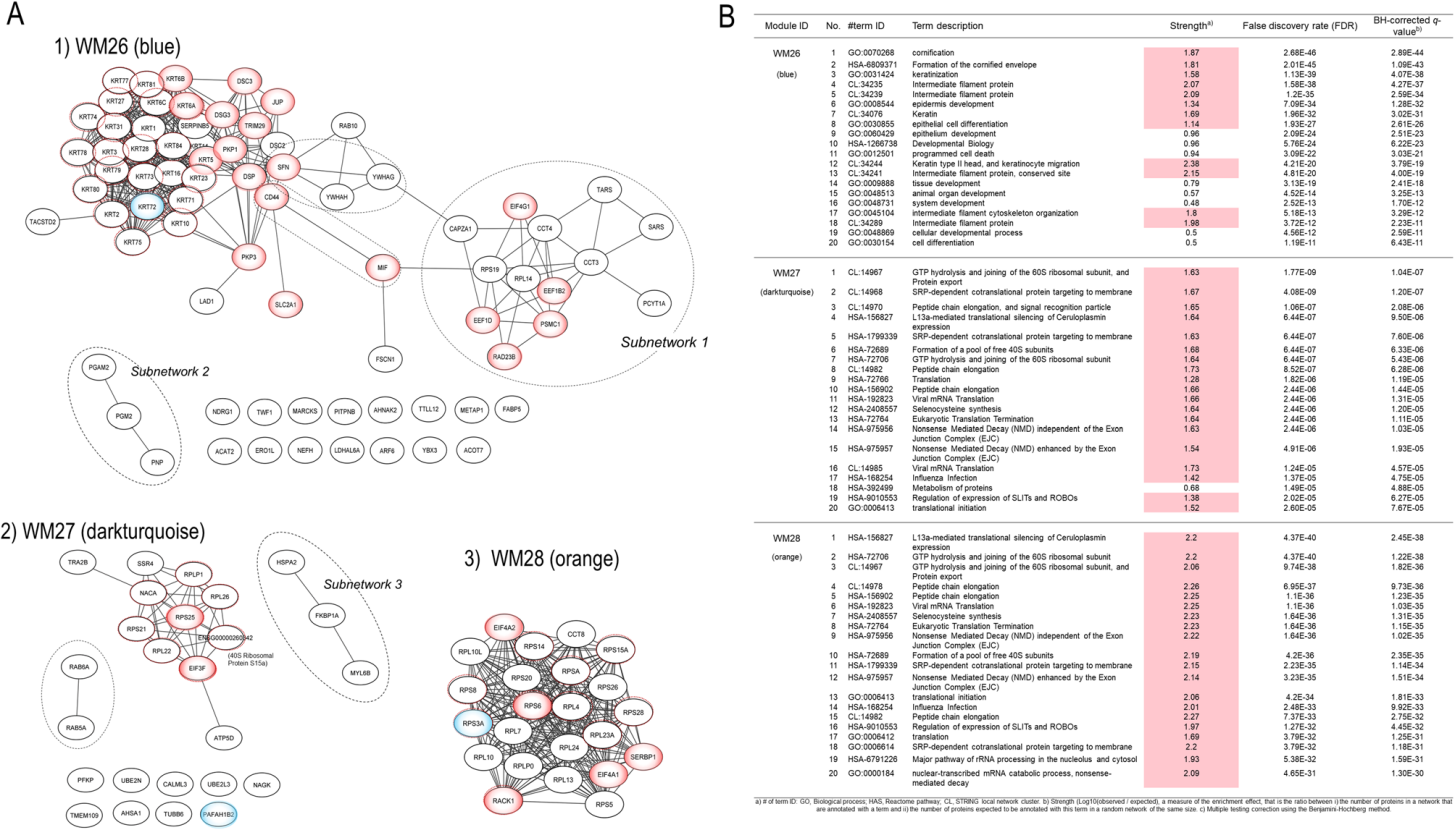
Data-driven protein co-expression networks and pathway enrichment results obtained for the lung SqCC trait. (**A**) The co-expression networks of respective modules: (1) WM26, (2) WM27, and (3) WM28 modules. Circle nodes in blue gradation and dotted red represent eigen proteins and/or hub proteins, respectively, for each module, and circle nodes in red gradation also indicate key proteins in the network modules. Dashed circles in black denote subnetworks and protein groups. (**B**) Among pathways enriched for the protein core networks obtained for biological process (GO) and Reactome pathways, the top 20 pathways are presented in the order of significance by the *q*-value. The dashed circles denote subnetworks and protein groups.

The biological associations between the proteins in each key protein network significant to SqCC were analyzed by mapping the network proteins in the human protein–protein interaction (PPI) network and by pathway enrichment (Fig. 3).

We used the Search Tool for the Retrieval of Interacting Genes/Proteins (STRING) database^11^ to generate the PPI networks for the three WGCNA network modules associated with SqCC–WM26, WM27, and WM28, and were reconstructed using the Cytoscape version 3.8.2 software program (Institute for Systems Biology, Seattle, WA, USA). Top hub proteins were determined using the cytoHubba plugin by maximal clique centrality (MCC)^12^. The three WGCNA modules, WM26 (*r* = 0.851, *q* = 0.0004), WM27 (*r* = 0.900, *q* = 7.34 × 10^–5^), and WM28 (*r* = 0.884, *q* = 0.0001), were significantly correlated with SqCC, where eigen proteins and/or hub proteins are indicated by blue and red dotted circles, respectively (Fig. 3). For the top three WGCNA modules significant to PPA (WM15, WM16, and WM18), their protein networks and pathways enriched are presented in Supplementary Fig. S3.

The three WGCNA protein network modules were significant to SqCC (Fig. 3A). The pathways enriched for WM26 (Fig. 3B) included (1) biological process (GO): cornification, keratinization, and intermediate filament cytoskeleton organization; (2) Reactome pathways: formation of the cornified envelope; and (3) STRING local network cluster: intermediate filament protein and keratin type II head and keratinocyte migration. Subnetwork 1 was significant to translation, in which key proteins and their associated pathways included EEF1B and EEF1D, eukaryotic translation elongation; CCT3 and CCT4, chaperonin-mediated protein folding; EIF4G1, signaling by insulin receptor; RAD23B, nucleotide excision repair; and PSMC1, cell cycle progression, apoptosis, or DNA damage repair. Translation initiation factor eIF4G was overexpressed in SqCC^13^. Subnetwork 2 is significant to the oxidoreduction coenzyme metabolic process and/or glycolysis and gluconeogenesis. The group of CD44 and MIF (macrophage migration inhibitory factor) suggests the negative regulation of DNA damage response and intrinsic apoptotic signaling pathway by the p53 class mediator. The 14-3-3 proteins, SFN (stratifin, 14-3-3 sigma, or epithelial cell marker protein 1), YWHAG (14-3-3 gamma), and YWHAH (14-3-3 eta), are involved in protein insertion into the mitochondrial membrane, which is involved in the apoptotic signaling pathway.

Niemira et al*.* conducted the RNA-seq based profiling of tissues obtained from 114 patients with NSCLC who received tumor resection surgery, followed by bioinformatics analyses combined with WGCNA^14^. Their GO enrichment analysis of genes differentially expressed in lung SqCC revealed deregulated processes of cornification, epidermis development, keratinization, and epidermal cell differentiation^14^, which is consistent with our results. Pak et al. investigated the morphologic characteristics of lung SqCC and concluded that keratinization in lung SqCC was associated with a poor prognosis and mostly with smoking^15^. The hub proteins in this module were the series of intermediate filament proteins including the eigen-protein KRT72 (keratin 72). KRT5 (keratin 5), KRT6A (keratin 6A), KRT6B (keratin 6B), DSG3 (desmoglein 3), and TRIM29 (tripartite motif-containing protein 29) are also highly connected proteins in this module, which were reported as potential biomarkers for distinguishing between SqCC and lung adenocarcinoma^16^. DSG3, a member of seven transmembrane desmosomal cadherins, was upregulated in various SqCC tissues, and its expression level correlated with clinical stages^17^. TRIM29, also known as the ataxia group D complementary gene (*ATDC*), is a transcriptional regulator involved in cell proliferation, differentiation, infiltration, migration, and invasion^18^. Expressions of TRIM29 were upregulated in numerous cancer types and were suggested to promote lung SqCC cell metastasis by regulating the autophagic degradation of E-cadherin^19^.

The enriched pathways of the WM27 module included (1) biological process (GO): translational initiation; (2) Reactome pathways: L13a-mediated translational silencing of ceruloplasmin expression, translation, and nonsense-mediated decay (NMD) independent of the exon junction complex; and (3) STRING local cluster: signal recognition particle-dependent cotranslational protein targeting the membrane, and peptide chain elongation (Fig. 3). Subnetwork 3 is significant for protein refolding, and the Ras-related proteins RAB5A and RAB6A are involved in cytosolic transport. The eigen-protein PAFAH1B2 (platelet-activating factor acetylhydrolase IB subunit alpha2, also known as the PAF-AH 30 kDa subunit) was overexpressed in some types of tumors including lung cancer^20^. The transcription of PAFAH1B2 is directly initiated by HIF1α activated in a hypoxic environment, and its overexpression induces epithelial–mesenchymal transition and subsequent aggressive phenotypes^21^. The hub proteins included the ribosomal proteins—RPLP1, RPL26, RPS25, RPS21, and RPL22, which participate in NMD, and EIF3F. Under cellular stress, translation initiation switches from cap-dependent translation to alternative mechanisms such as internal ribosome entry site (IRES) initiation^22^. RPS25 is the key ribosomal protein mediating c-MYC IRES-dependent translation under endoplasmic reticulum stress^23^. EIF3F (eukaryotic translation initiation factor 3F) was overexpressed in lung cancer cells, which were reported to reprogram cell proliferation and energy metabolism^24^.

The enriched pathways of WM28 (Fig. 3) included (1) biological process (GO): translational initiation, nuclear-transcribed mRNA catabolic process, and NMD; (2) Reactome pathways: L13a-mediated translational silencing of ceruloplasmin expression, GTP hydrolysis, and joining of the 60S ribosomal subunit, peptide chain elongation, and NMD independent of the exon junction complex; and (3) STRING local network cluster: GTP hydrolysis and joining of the 60S ribosomal subunit and protein export, and peptide chain elongation. Thus, WM27 and WM28 shared almost the same enriched pathways. The hub proteins in this module were the ribosomal proteins including the eigen-protein RPS3A. The expression of RPS3A was upregulated, and its frequent enhancement was reported in patients with SqCC^25^. RACK1 (receptor for activated C kinase 1) is a member of the 40S ribosomal subunit involved in translational repression and the initiation of ribosome quality control via the regulatory ubiquitylation of 40S ribosomal proteins^26^. SERBP1 (SERPINE1/PAI1 mRNA-binding protein 1, also known as PAI-RBP1), a member of the serine protease inhibitor, was identified as a partner of RACK1^27^. SERBP1 was overexpressed in various cancers including breast cancer, ovarian carcinoma, glioblastoma, and also lung SqCC, and this might be associated with tumorigenicity and resistance to anticancer drugs^28^. RPS6 (40S ribosomal protein S6), a substrate for p70S6 kinase (p70S6K), is known to play important roles in tumorigenesis and development. The highly expressed RPS6 and its phosphorylated form have been observed in various cancers including NSCLC, where upstream of Akt2/mTOR/p70S6K signaling pathway was aberrantly regulated^29^. The dephosphorylation of RPS6 can inhibit the mTOR pathway, resulting in the inhibition of tumor growth and metastasis^30^. Eukaryotic translation initiation factors, EIF4A1 and EIF4A2, are the RNA helicases belonging to the EIF4F initiation complex that unwind mRNA during translation^31^. EIF4A1was upregulated in various cancers while the downregulation of EIF4A2 in NSCLC was associated with a poor prognosis^32^.

### Multivariate correlation analysis of semiquantitative key protein expressions

**Figure 4:**
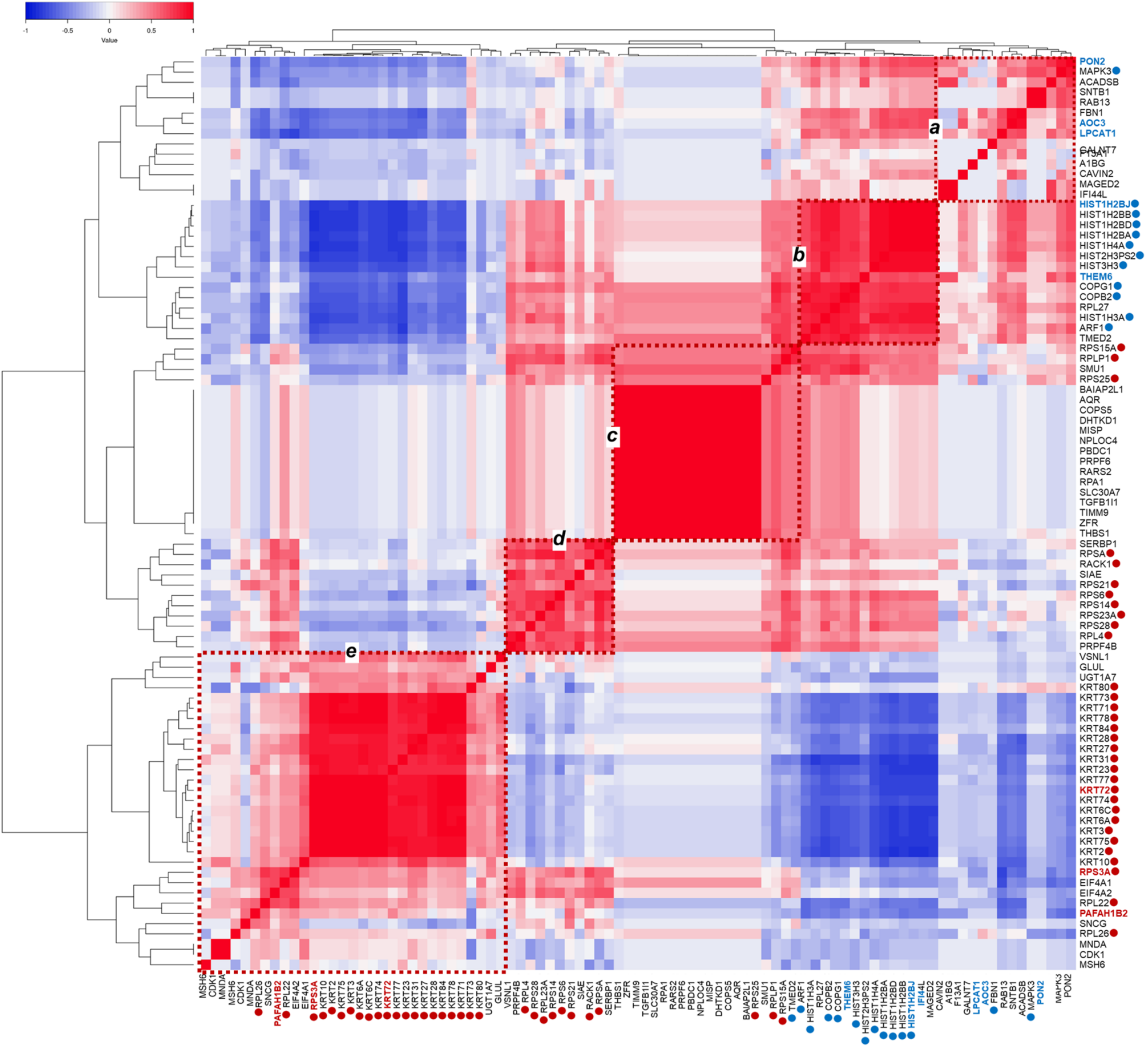
Multivariate correlation analysis for the spectral counting-based expression of 89 eigen proteins and/or hub proteins and other key proteins expressed among all the modules identified for both the SqCC and PPA traits. Clusters are denoted by *a*, *b*, *c*, *d*, and *e*. Eigen proteins in the WGCNA network modules significant for SqCC or PPA are denoted in red or blue letters, respectively; the hub proteins are also indicated by red or blue-filled circles, respectively.

Representative proteins expressed throughout all the 30 modules were subjected to multivariate correlation analysis (MVA). As a result, the spectral count-based semiquantitative expression of 89 key proteins including eigen proteins and/or hub proteins was clustered into several groups (*a*, *b, c*, *d*, and *e*; Fig. 4). The clusters *c*, *d,* and *e* were characteristic of the SqCC trait whereas the clusters *a* and *b* were characteristic of the PPA trait. Of these, cluster *e* included hub proteins of the WM26 module and eigen proteins of the SqCC characteristic modules. Cluster *d* included most of the hub proteins in WM28.

### Causal networks, canonical pathways, and downstream regulator effects predicted by IPA

Causal network analysis together with downstream annotation was performed for the WGCNA modules significant to SqCC, using the Ingenuity Pathway Analysis (IPA) (http://www.ingenuity.com) software^33^. Table 2 briefly summarizes the top master regulators, diseases or functions, and canonical pathways predicted for the three SqCC-characteristic WGCNA modules. Master regulators were predicted to be activated or inhibited (|*z*-value|> 2.0) and upregulated (1.5 < *z*-value < 2.0) with the significance of network bias-corrected *p*-value < 0.005. The top master regulators with high values in activation or inhibition score (*z*-score) in causal networks, which were significantly associated with SqCC (WM26, WM27, and WM28) and PPA (WM15, WM16, and WM18), are presented in Supplementary Tables S1 and S2, together with their participating regulators and target molecules in the datasets.

**Table 2:** Representative master regulators predicted to be activated or inhibited (|z-value|> 2.0) and upregulated (1.5 < z-value < 2.0) are summarized for the three WGCNA modules significant to lung SqCC (network bias-corrected *p*-value < 0.005), in which top annotations on canonical pathways, diseases or functions, and downstream regulatory effects are also provided.

Module ID (color)	Causal networks		Canonical pathways		Diseases or functions			Downstream regulatory effects	
	Master regulators	z-score	Top annotations (z-value)	*p*-value	Top annotations	*p*-value	(z-value)	Top annotation	Consistency
WM26 (blue)	*MNK1/2*	3.638	Glucocorticoid receptor signaling	5.01E−24	Keratinization of epidermis	2.46E−52		Cell proliferation of squamous cell carcinoma cell lines	3.795
	*ROCK2*	2.646	HIPPO signaling (− 1)	1.78E−04					
	*EFNA4, EFNA3, EFNA5, EFNA2*	2.449	Purine ribonucleosides degradation to ribose-1-phosphate	2.95E−04					
	*EFNA1*	2.236	Cell cycle: G2/M DNA damage checkpoint regulation	5.62E−04					
	Desmoplakin, *DSP*	2	ERK5 signaling	1.74E−03					
	*SFN*	2	p53 signaling	4.17E−03					
	Tumor protein 63 (*TP63*)	1.897	IGF-1 signaling	4.90E−03					
	Max dimerization protein 1 (*MXD1*)	− 2.132							
	*KMT2D*	− 2.828							
	*CDK4/6*	− 2.828							
WM27 (darkturquoise)	Zinc finger E-box-binding homeobox (*ZEB*)	2.714	EIF2 signaling	1.26E−07	Initiation of translation of protein	9.92E−09		N/A	
	Carbohydrate-responsive element-binding protein (*MLXIPL*)	2	mTOR signaling	7.59E−05	Nonsense-mediated mRNA decay	1.22E−07			
	*cdk*	2	Regulation of eIF4 and p70S6K Signaling	7.76E−04	Metabolism of protein	1.12E−05	(− 0.436)		
	Baculoviral IAP repeat-containing protein (*BIRC5*)	1897	Remodeling of Epithelial Adherens Junctions	2.51E−03					
	*FABP2*	1.89							
	*MYCN*	1.633							
	Rapamycin-insensitive companion of mTOR (*RICTOR*)	− 2							
	5-Fluorouracil(5-FU), chemical drug intervention	− 2							
	La-related protein 1 (*LARP1*)	− 2.24							
	*Mir200*	− 2.714							
WM28 (orange)	*MYC*	4	EIF2 signaling	2.51E−35	Initiation of translation of protein	5.31E−39		Cell death of osteosarcoma cells	8.132
	*MLXIPL*	3.873	Regulation of eIF4 and p70S6K signaling	2.00E−20	Nonsense-mediated mRNA decay	1.31E−35			
	*MYCN*	3.742	mTOR signaling	3.16E−19	Translation of protein	4.18E−31			
	*TCR*	2.449	HIF1α signaling	1.78E−02	Metabolism of protein	1.81E−21	(− 1.72)		
	*CAMK2N2*	− 2.138	Insulin secretion signaling pathway	2.51E−02	Cell death of osteosarcoma cells	5.88E−13	(− 2.828)		
	CD 437, chemical drug intervention	− 2.236			Translation of mRNA	2.65E−11			
	ST1926, chemical drug intervention	− 2.449			Cell death of tumor cells	2.28E−10	(− 2.53)		
	*RICTOR*	− 3			Cell death of cancer cells	1.09E−09	(− 2.333)		
	Sirolimus (Rapamycin), chemical drug intervention	− 3.464							
	La-related protein 1 (*LARP1*)	− 3.873							

#### Master regulators predicted for the WM26 module

Highly activated master regulators predicted for the WM26 module were *MNK1/2*, *ROCK2*, *EFNA4*, *EFNA3*, *EFNA5*, *EFNA2*, *DSP*, *EFNA1,* and *SFN*, while *KMT2D* and *MXD1* were highly inhibited. *MNK1/2* encodes mitogen-activated protein kinases interacting protein kinases 1 and 2 (Mnk1 and Mnk2) which are known to play important roles in controlling signals involved in mRNA translation. *ROCK2* encodes the Rho-associated coiled-coil-containing protein kinase 2 (also known as Rho-kinase 2 or ROCK-II), belonging to mammalian serine/threonine kinases and downstream effectors of the small GTPase RhoA, which are key regulators of keratinocyte adhesion and terminal differentiation^34^. ROCK2 is an oncoprotein that acts as a prognostic marker in various solid tumors^35^.

EFNA4, EFNA3, EFNA5, EFNA2, and EFNA1 are members of the Eph (erythropoietin-producing hepatoma or Ephrin) receptors of the A-type, which are the most important family of receptor tyrosine kinases involved in signaling pathways of embryogenesis and tissue patterning. Eph signaling regulates cell morphology and migration by modifying cell adhesion and organizing actin cytoskeleton and then affects cell proliferation and differentiation^36^. Eph receptors are expressed in cancer cells and the tumor microenvironment involved in tumorigenesis and metastasis^37^. However, Eph receptors can act both as tumor promoters and suppressors, depending on the cancer type^37,38^. Regarding lung cancer, the upregulation or overexpression of EFNA1, EFNA2, EFNA4, EFNA5, and EFNA7 are indicative of tumor-promoting roles in lung cancer^39^.

*KMT2D* encodes histone-lysine *N*-methyltransferase 2D, formerly named MLL2 (myeloid/lymphoid or mixed-lineage leukemia 2), which methylates “Lys-4” of histone H3 (H3K4me), inducing epigenetic transcriptional activation. This epigenetic regulator *KMT2D* is the most frequently mutated in all cancers, and its mutations are notably associated with keratinocyte cancers^40^. Lin-Shiao et al. recently reported that KMT2D interacts with the transcription factor TP63 on chromatin and regulates TP63 target enhancers to coordinate epithelial homeostasis and GO analysis for genes upregulated in shKMT2D-treated keratinocytes showed epithelial cornification, keratinization, differentiation, and development as the most enriched category^41^.

Tumor protein 63 (TP63), the master regulatory transcription factor of epithelial tissues, was predicted to be upregulated (*z*-value = 1.897). TP63 regulates most of the same target genes involved in vitamin D and retinoid signaling that are regulated by KMT2D^41^. Vitamin D receptor regulates the c-MYC/MXD1 network, in which the transcriptional repressor MXD1 is the antagonist, to suppress c-MYC function, preventing epidermal tumor formation^42^. Interestingly, the MXD1 causal network inhibited in this study suggested the activation of c-MYC.

*SFN* was activated, encoding SFN, which is a cell cycle checkpoint protein and is present mainly in tissues enriched in the stratified squamous keratinizing epithelium. SFN binds to translation and initiation factors, and especially stimulates tumor initiation and the progression of early-stage lung adenocarcinoma^43^. *DSP* (desmoplakin) encodes the major high-molecular-weight protein of desmosomes. Desmosomal genes are expressed differently between lung adenocarcinoma and lung SqCC although the mechanism regulating their expression remains unknown.

The protein networks of the WM26 module demonstrated the involvement of upregulated desmosomal proteins including DSP, DSC2 (desmocollin 2), DSC3 (desmocollin 3), JUP (junction plakoglobin), PKP1 (plakophilin 1), and PKP3 (plakophilin 3). Martin-Padron et al. reported that PKP1 was overexpressed and increased cell proliferation and cell survival in lung SqCC, and found that PKP1 enhances *MYC* translation together with the translation initiation complex by binding to the MYC mRNA^44^. Kudo et al. demonstrated via immunohistochemical staining that the expression of *DSC3*, *SFN*, *DSP*, and *JUP* among cell adhesion and growth inhibitor genes was highly increased in *TP53*-mutated tumors and that *TP53*-mutated tumors exhibited high nuclear staining of the TP53 protein only in tumor cells at the tumor margins adjacent to the stroma but not in the tumor interior; thus, exhibiting tumor cell heterogeneity in the expression of mutated *TP53* protein between the tumor interior and margins^45^.

Keratinization of the epidermis and cell proliferation of SqCC cell lines were thus significantly annotated to the WM26 network module (Table 2), with key regulators being *HIF1A* (hypoxia-inducible factor 1 subunit alpha) and *IGF1* (insulin-like growth factor 1).

#### Master regulators predicted for the WM27 and WM28 modules

The WM27 and WM28 modules were found to share the same master regulators that representatively included highly activated *MLXIPL* and *MYCN*, and highly suppressed *LARP1* and *RICTOR* (rapamycin-insensitive companion of mTOR), which were remarkable in the WM28 module: *LARP1*, overlap *p*-value = 3.59 × 10^–32^ and *z*-value = − 3.873; *MLXIPL*, overlap *p*-value = 8.54 × 10^–30^ and *z*-value = 3.873; *MYCN*, overlap *p*-value = 3.27 × 10^–22^ and *z*-value = 3.742; *RICTOR*, overlap *p*-value = 4.10 × 10^–12^ and *z*-value = − 3.0.

Highly activated *ZEB* and highly inhibited *Mir200* were characteristic of the WM27 module. *ZEB* encodes the zinc finger E-box-binding homeobox proteins ZEB1 and ZEB2 which regulate the epithelial–mesenchymal transition pathway as both transcriptional activators and repressors. The miRNA-200 (*miR-200*) family can repress ZEB proteins to regulate epithelial differentiation^46^. Activated *ZEB* and inhibited *Mir200* suggested progressive tumorigenesis acquiring a mesenchymal phenotype in lung SqCC. The high expression of ZEB1 is associated with tumor grade in NSCLC or distant metastasis in lung SqCC^47^.

LARP1 (La-related protein 1) is an RNA binding protein and mTORC1 effector involved with terminal oligopyrimidine (TOP) mRNA translation. Surprisingly, LARP1 was found to be highly suppressed in this study. Significant upregulated LARP1 was frequently reported to correlate with adverse prognosis in several cancers including NSCLC^48^. In contrast, clear cell renal cell carcinoma progression was promoted by decreased LARP1 derived from the downregulation of the long noncoding RNA ASB16-AS1 by inhibiting miR-185-5p and miR-214-3p^49^. Our result should be understood along contexts underpinning disease mechanisms and because the function of LARP1 is highly controversial^50^.

The highly activated *MYC*, *MLXIPL*, and *MYCN* and highly suppressed *LARP1* and *RICTOR* were predicted for the WM28 module. *MLXIPL* encodes a carbohydrate-responsive element-binding protein, which is a basic helix-loop-helix leucine zipper (bHLH-LZ) transcription factor of the MYC/MAX/MAD superfamily, promotes aerobic glycolysis through inhibition of TP53, resulting in tumor cell proliferation^51^.

*MYCN*, a member of the *MYC* family of oncogenes, also encodes a bHLH-LZ protein MYCN, and its deregulation was reported in various cancer types and was often associated with a poor prognosis. *MYCN*-amplified cancer cells exhibit the enhanced expression of genes and proteins involved in aerobic glycolysis (referred to as the Warburg effect), oxidative phosphorylation, and the detoxification of reactive oxygen species (ROS)^52^.

Numerous chemical drug interventions including sirolimus (rapamycin) were inhibited (Table 2), suggesting an involvement of potential therapeutic targets in those data-driven networks. The translation initiation of protein, nonsense-mediated mRNA decay, and negatively regulated cell death of tumor and cancer cells were annotated to the WM28 module (Table 2).

Interestingly, the EIF2 signaling pathway exhibited the highest significance in overlapping *p*-values among the top canonical pathways predicted for both the WM27 and WM28 network modules, and it was remarkably activated especially for WM28 (overlap *p*-value = 2.77 × 10^–35^ and *z*-value = 2.828). The upregulation of eIF2α and eIF2β, which are members of translation initiation factors (eIFs), has been reported in several cancer types including lung cancer, and is often associated with poor prognosis in patients^53^. Bilguun et al. found that STXBP4 (syntaxin binding protein 4) which targets TP63 was crucially associated with lesion growth in lung SqCC patients, in which the eIF2 signaling pathway was the most significantly activated^54^.

The top canonical pathways predicted for the three SqCC characteristic modules are also listed in Table 2. The WM26 module was most significantly annotated to glucocorticoid receptor signaling and inhibited HIPPO signaling, and the WM27 and WM28 were significant to eIF2 signaling, mTOR signaling, and the regulation of eIF4 and p70S6K signaling.

### Genomic alteration landscape of early-stage SqCCs based on the TCGA database

Kim et al. performed a comparative genomic analysis between Korean and North American lung SqCC samples and demonstrated a spectrum of genomic alterations similar to the two ethnically different cohorts, which contrasts with the differences noted in lung adenocarcinoma^55^. The TCGA lung SqCC sub-datasets (T1A-T2A: *n* = 184) were selected to match our early tumor stage patients’ group, and their genomic alteration profiles were visualized using the cBioPortal Pan-Lung cancer (TCGA, Cell 2018) (https://www.cbioportal.org/) (Supplementary Fig. S4). Frequently altered driver mutation candidates were as follows: *TP53* (80%) and *CDKN2A* (40%) in cell cycle; *PIK3CA* (43%), *PTEN* (19%), and *FGFR1* (17%) in mitogenesis and RAS signaling; *SOX2* (39%) and *TP63* (31%) in squamous cell differentiation; *KMT2D* (23%) and *FAT1* (20%) in transcription and gene expression; and *SYNE1* (29%) and *NFE2L2* (21%) in cell survival. Characteristics regarding mRNA-level expressions types of genomic alterations represent *PIK3CA*, frequent missense (driver) and high amplification; *PTEN*, frequent truncating (driver) and missense (driver); *SOX2* and *TP63*, high amplification; *KMT2D*, highly frequent truncating (driver) and splice (driver); and *NEF2L2*, frequent missense (driver) (Supplementary Fig. S5). Shang et al*.* reported a mutational landscape of Chinese lung SqCC patients, in which mutation frequencies of *PIK3CA*, *NFE2L2*, and *KMT2D* were most significant^56^, which was quite similar to our TCGA-based study, although most of their cohorts were at advanced tumor stages. In our TCGA-based genomic alterations, *PIK3CA*, *SOX2*, and *TP63* co-occurred with high significances of *q*-value < 0.001, and *NOTCH2* and *DDR2* co-occurred with *q* = 0.002. No driver mutation candidates were mutually exclusive. *TP63* was co-expressed with *PKP1*, *KRT6A*, *DSG3*, *KRT6C*, *NFE2L2*, *KRT6B*, and *SOX2* at the mRNA level (Spearman’s correlation > 0.6 and *q*-values < 5.0 × 10^–18^), which was found to be consistent with co-expression networks of the WM26 module (Fig. 2A). Our IPA analysis of the WM26 module annotated the proliferation of SqCC, in which the key regulator *HIF1A* was important in the adaptive response to hypoxia and angiogenesis. The ROS-responsive transcription factor NRF2 encoded by *NEF2L2* can bind and transactivate an antioxidant response element upstream of *HIF1A*, through which the expression of HIF1α encoded by *HIF1A* is regulated directly by NRF2^57^. Moreover, the accumulation of HIF1α directly upregulates SLC2A1 (known as GLUT1, glucose transporter-1), as we indeed observed in the co-expression networks of WM26, most likely suggesting hypoxia-induced metabolic changes to aerobic glycolysis (the Warburg effect).

### Overview, limitations, and conclusion

**Figure 5:**
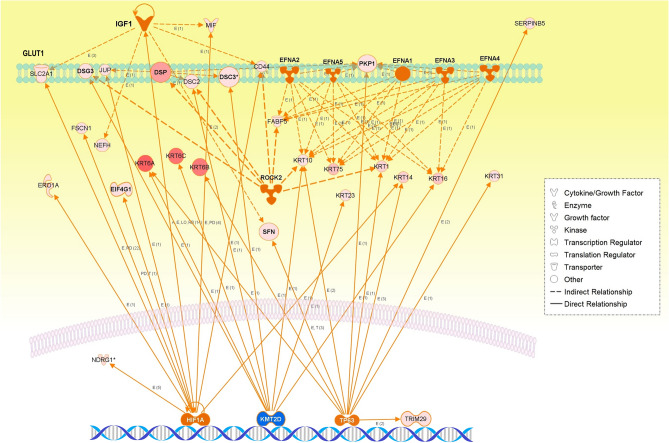
The integrative networks of representative master and participating regulators predicted for the WM26 module.

We identified protein co-expression networks significantly associated with lung SqCC by WGCNA following MS-based proteomic analysis. Multivariate correlation analysis for semiquantitative expressions of key proteins exhibited protein clusters characteristic of the SqCC trait, which were well differentiated from those characteristics of the PPA trait. Strikingly, pathways enriched for the WM26 module predominantly involved keratinization. The predicted causal networks were also annotated to cell morphology and keratinization of the epidermis, in which key master regulators were highly activated *ROCK2* and *Ephrs* (*EFNA1-5*), while an epigenetic regulator and lung tumor suppressor *KMT2D* were highly inhibited. Downstream regulator effects were annotated to the cell proliferation of SqCC, and a key regulator, *HIF1A*, was involved in hypoxia-induced metabolic changes to aerobic glycolysis. Correspondingly, upregulated *TP63* was predicted for the WM26 module (Table 2 and Fig. 5), and genomic alterations in the early-stage TCGA SqCC database exhibited highly amplified and frequently truncated driver mutations of *KMT2D* (Supplementary Fig. S5).

**Figure 6:**
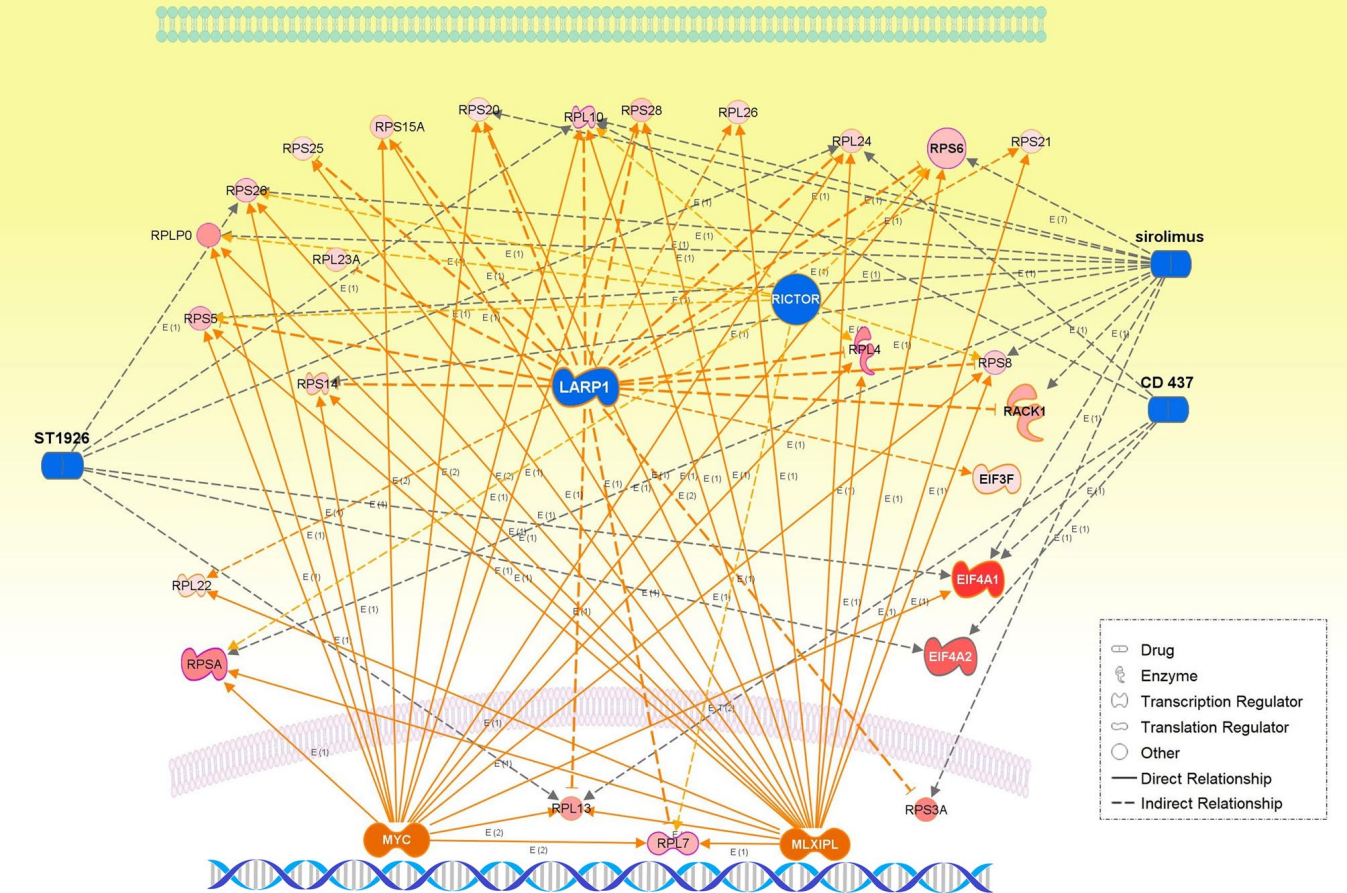
The integrative networks of representative master and participating regulators predicted for the WM27 and WM28 modules, together with chemical drug interventions (CD 437, ST1926, and sirolimus [rapamycin]).

Pathways enriched and master participating regulators of causal networks predicted for both the WM27 and WM28 modules were annotated to translation initiation and nonsense-mediated mRNA decay. The co-expression networks of the WM27 module were characteristic of mesenchymal transformation by activated ZEB, and of upregulated hub ribosomal proteins including the key ribosomal protein RPS25 of IRES-dependent translation. Hong et al*.* revealed that non-phosphorylated LARP1 interacts with ribosomal protein mRNAs and inhibits their translation while LARP1 phosphorylated by mTORC1 and Akt/S6K1 allows ribosomal protein mRNA translation^58^. However, this switching mechanism of translation of ribosomal protein mRNAs depending on phosphorylation/non-phosphorylation of LARP1 does not seem to interpret our results of both the highly suppressed LARP1 and upregulated expressions of the hub ribosomal proteins including RACK2, RPS6, and RPS25, which were predicted to be indirectly targeted by LARP1. Suppression of the cell death of tumor and cancer cells was centrally annotated as downstream regulator effects to the WM28 module, in which key master regulators were highly suppressed *LARP1* and highly activated *MYC* and *MLXIPL* (Table 2 and Fig. 6). Even more, interestingly, the eIF2 signaling was annotated commonly to both two modules with the highest significance.

Collectively, all the results we obtained in this study might allow a possible scenario as follows. Physiological stressors such as hypoxia and/or ROS downregulate mTOR activity which represses cap-dependent translation and reduces overall protein synthesis, in which the eIF2 ternary complex mostly plays an important role^59^. The inhibition of protein synthesis leads to the activation of alternative cap-independent translation of mRNA subsets which utilizes IRESs located in the 5′ untranslated region of mRNA^60^. These mRNAs encode oncogenic proteins such as HIF1α, MYC, c-MYC, VGFA, and BCL-2, which promote the progression of tumorigenesis, angiogenesis, and cancer cell survival. Thus, the eIF2 complex assembly importantly functions in switching from cap-dependent to cap-independent translation^60,61^. This might explain why LARP1, the master regulator in the cap-dependent TOP mRNA translation, was highly suppressed and why this loss of LARP1 caused the reduction of mTOR activity and downstream mTORC1 signaling including RICTOR.

The limitation of this study was the number of patients examined. We plan to validate the data-driven protein networks as systems obtained from this study using a larger sample size of the external cohort in the future. Numerous immunohistochemical (IHC) studies have already been reported for several member proteins of the WM26 module such as DSG3, CK5(KRT5), and TRIM29 (Fig. 3A), and p63 [TP63, one of the master regulators predicted for the WM26 network module (Table 2)], which are often clinically used to diagnose lung SqCC^16,62^. Many studies have shown that p63 is a sensitive (90%) and fairly specific marker for lung SqCC, and tripartite motif-containing 29 (TRIM29) is a sensitive marker (93.7%) for lung SqCC and is a fairly specific marker staining only 6.1% of lung adenocarcinomas (https://biocare.net/product/p63-trim29-antibody/). Desmoglein 3 (DSG3) is often highly expressed in various squamous cell carcinomas (SqCC) and has demonstrated a sensitivity of 85–99% and an ability to discriminate lung adenocarcinoma with a specificity of 98–100%. A cocktail of DSG3 + CK5 antibodies reported sensitivities of 93% and 100% for lung SqCC, with a specificity of 100% against lung adenocarcinoma (https://biocare.net/product/desmoglein-3-ck5-antibody/).

In conclusion, we successfully applied WGCNA to clinical proteomic datasets. Our results could identify data-driven network profiles and their master regulators characterizing cancerous cells microdissected from FFPE tissues of lung SqCCs, exhibiting the progression of SqCC concomitantly with aberrant keratinization, epithelial-mesenchymal transformation, aerobic glycolysis, and negatively regulated cell death. Collectively, the results obtained in this study suggest an underlying disease mechanism of lung SqCC progression caused largely by switching to the cap-independent, IRES-dependent translation of mRNA subsets encoding oncogenic proteins. We plan to conduct a larger-sample cohort study, including genomic alteration analysis, to investigate data-driven proteogenomic networks, which will further provide clinically important information on the proteogenomic landscape of lung SqCCs.

## Materials and methods

### FFPE tissue specimens and sample preparation

Among 1333 patients who underwent surgical lung cancer resection at St. Marianna University Hospital between 2000 and 2020, only 198 (14.9%) had tumors that were histologically confirmed lung SqCC. The pathological specimens were independently reviewed by two pathologists (H. N. and M. T.) to confirm that they satisfied the 2015 World Health Organization classification criteria for lung tumors (histological criteria)^63^. FFPE tumor tissue blocks from 15 surgical specimens histologically confirmed as lung SqCC and PPA were obtained without patient identifiers from the St. Marianna University School of Medicine Hospital. Informed consent was obtained from all participating subjects. The protocol was approved by the Institutional Review Board of St. Marianna University School of Medicine (approval no. 1461) and the study adhered to the Helsinki Declaration. For tissue microdissection, 10-μm-thick sections from the FFPE tumor blocks were cut and placed on DIRECTOR slides (OncoPlex Diagnostics Inc., Rockville, MD, USA). The sections were deparaffinized and stained with hematoxylin using standard histological methods before dissection. Microdissection was performed using a Leica LMD7 microdissection microscope (Leica, Wetzlar, Germany). A total area of 4 mm^2^ with approximately 15,000 tumor cells was directly transferred from the FFPE sections via laser dissection into the cap of a 200-μL low-binding tube (Supplementary Fig. S1).

Proteins were extracted and digested with trypsin using the Liquid Tissue MS Protein Prep kits (OncoPlex Diagnostics Inc.) according to the manufacturer’s protocol^64^. Briefly, dried microdissection pellets were suspended in 20 μL of Liquid Tissue buffer and heated at 95 °C for 90 min followed by cooling on ice, at which point 0.1 μg of trypsin was added to each tube. The tubes were then incubated at 37 °C for 18 h. The digested samples were dried and then resuspended in 50 μL of 2% acetonitrile aqueous solution containing 0.1% trifluoroacetic acid. Finally, the digested samples were frozen at − 80 °C until further processing.

### Liquid chromatography-tandem MS (LC–MS/MS)-based proteomic analysis

A label-free quantitation approach using spectral counting by LC–MS/MS was adopted for the global proteomic analysis. The digested samples (5 μL for a single run) were analyzed in triplicate by LC–MS/MS using a reverse-phase LC system interfaced with a Q Exactive Orbitrap mass spectrometer (Thermo Fisher Scientific, Bremen, Germany) via a nano-electrospray ionization device (AMR Inc., Tokyo, Japan). The LC system consisted of an Ultimate3000 HPLC System (Thermo Fisher Scientific), a trap cartridge (0.3 mm × 5.0 mm, CERI, Tokyo, Japan), and a capillary separation column (Zaplous column alpha-PepC18, 3 μm, 12 nm, 0.1 mm × 150 mm, AMR Inc.) fitted with an emitter tip (FortisTip, OmniSeparo-TJ, Hyogo, Japan). An auto-sampler (HTC-PAL, CTC Analytics, Zwingen, Switzerland) loaded an aliquot of samples into the trap, which was then washed with solvent A (2% acetonitrile aqueous solution containing 0.1% formic acid) to concentrate the peptides in the trap and desalt them. Subsequently, the trap was connected in series to the separation column, and the peptides were eluted from the whole column with 0.1% formic acid aqueous solution and acetonitrile by linear 5–40% acetonitrile concentration gradient over 90 min at a flow-rate of 500 nL min^−1^. LC–MS/MS analysis and protein identification have been described in detail previously^65,66^. In brief, the raw data were processed using PatternLab for Proteomics software v4.0. Peptide sequence matching was performed using the Comet algorithm against the UniProt Homo sapiens database. A target-reverse strategy was employed for increased confidence in protein identification. This search considered tryptic peptide candidates, and the formylation of lysine and oxidation of methionine were considered as variable modifications. The Comet search engine considered a precursor mass tolerance of 40 ppm and a fragment bin tolerance of 0.02. The validity of the peptide spectrum matches was assessed using PatternLab’s Search Engine Processor (SEPro) module. Acceptable FDR for spectra, peptide and protein are 3%, 2%, and 1%, respectively. The expressions of the identified proteins were assessed by spectral count-based protein quantification. The spectral count is the number of MS/MS spectra assigned to each protein.

### WGCNA

The similarity in the protein expression patterns was calculated for all protein pairs using their pairwise Pearson’s correlation coefficient. An adjacency matrix is computed by increasing the similarity matrix up to the power of 100 to obtain a co-expression network with scale-free properties. A topological overlap matrix (TOM), which considers topological similarities between a pair of proteins in the network, was then generated from the resultant scale-free co-expression network. We generated a tree that clustered proteins in its branches by hierarchical clustering using the dissimilarity according to TOM (1 − TOM), and protein modules were determined using dynamic tree cutting to trim the branches^9^.

Modules were summarized by the first principal component referred to as eigen protein in the text, which has the highest connectivity in the module. Module membership, defined as the correlation between the protein expression profile and the module eigen protein, was measured with values ranging from 0 to 1, with 0 representing a gene that is not part of the module, and 1 representing high connectivity with the module. Subsequently, the module-trait association was determined using the correlation between the module eigen protein and the two clinical traits, SqCC and PPA. A protein module was summarized by the top hub protein (referred to as eigen protein) with the highest connectivity in the module. To identify the protein modules associated with clinical traits, we calculated the correlation coefficients between the eigen proteins and clinical traits. WGCNA analysis was conducted using a Garuda Platform Gadget (The Systems Biology Institute, Tokyo, Japan) that implemented the WGCNA pipeline based on the WGCNA R-package^9^.

### Protein–protein interaction network construction

To construct a protein interaction network for a protein module, we used the STRING database (version 11.0)^11^. STRING networks were calculated under the criteria for linkage with experiments, databases, text mining, and co-expression alone, with the default settings (medium confidence score: 0.400 network depth: 0 interactions). Functional enrichment results were obtained for canonical pathways, with *p* < 0.05. Proteins in a protein module were mapped in the protein interaction network from the STRING database, to produce the results of the enrichment analysis regarding the biological process (GO), STRING local network cluster (CL), and Reactome pathways (HAS). The enrichment effect was also evaluated by the measure, Strength, which is the ratio (log_10_ [observed/expected] regarding (i) the number of proteins in a network annotated with a term and (ii) the number of proteins expected to be annotated with this term in a random network of the same size. Subsequently, protein networks imported from the STRING database were visualized using Cytoscape version 3.8.2. The proteins inside co-expression modules exhibit high connectivity, and the proteins within the same module may play similar roles. We identified hub proteins in each module according to their intramodular connectivity and correlation with module eigen proteins. The top 20 high-degree proteins were identified using the cytoHubba plugin^12^. The top-ranked proteins in each module were considered to be hub proteins—“highly connected proteins.” Functional enrichment results were obtained for canonical pathways, considering *p* < 0.05 to be statistically significant. Multivariate correlation analysis of semiquantitative key protein expressions was performed using the JMP software (SAS Institute, Cary, NC, USA).

### Master regulator and causal network analysis and downstream regulator effect annotation predicted by IPA

Canonical pathways, master regulators, and causal networks were predicted using the ingenuity pathway analysis (IPA) software^33^. Quantile-normalized protein expression data for the selected modules were used as input data sets. Causal networks (*p* < 0.005) were predicted from the WGCNA network modules significantly associated with the two clinical traits (SqCC and PPA), where the activation and inhibition of a predicted network were defined by *z*-values > 2.0 and <  − 2.0, respectively. Upregulation and downregulation were also defined as 1.5 < *z*-value < 2.0 and − 2.0 < z-value < − 1.5, respectively.

## Supplementary Information

Supplementary Information.

## Data Availability

The unfiltered MS datasets generated and analyzed in this study have been deposited in the ProteomeXchange (http://proteomecentral.proteomexchange.org) and jPOST with the dataset identifiers PXD027381 and JPST001260, respectively.
