# Supplementary material for: Protein co-expression network-based profiles revealed from laser-microdissected cancerous cells of lung squamous-cell carcinomas
Source: Sci Rep. 2021 Oct 12;11:20209. doi: 10.1038/s41598-021-99695-x (PMC8511190; doi:10.1038/s41598-021-99695-x)

Supplementary Information File 1

Protein Co-expression Network-based Profiles Revealed from Laser-microdissected Cancerous Cells of Lung Squamous-Cell Carcinomas

Toshihide Nishimura*, Kiyonaga Fujii, Haruhiko Nakamura, Saeko Naruki, Hiroki Sakai, Hiroyuki Kimura, Tomoyuki Miyazawa, Masayuki Takagi, Naoki Furuya, Gyorgy Marko-Varga, Harubumi Kato and Hisashi Saji

Table S1. The top master regulators and participating regulators of the three network modules (WM26, WM27, and WM28) significant to the lung SqCC, together with target molecules in the datasets, which were predicted to be activated or inhibited (|z-value|> 2.0) and up-regulated (1.5 < *z*-value < 2.0) with the significance of network bias-corrected *p*-value < 0.005. Chemical drugs are indicated in red letter.

Module ID	*Master Regulator*	Molecule Type	*Participating regulators*	Depth	Predicted Activation	Activation *z*-score	*p*-value of overlap	Network bias-corrected *p*-value (<0.005)	Target molecules in Dataset
WM26	ROCK2	kinase	ROCK2	1	Activated	2.646	3.41E-11	0.0001	CD44,DSC2,DSG3,DSP,FABP5,KRT1,KRT10
	MXD1	transcription regulator	CCND1,MXD1,MYC	2	Inhibited	-2.132	4.69E-11	0.0001	CCT3,CD44,DSP,EEF1B2,EIF4G1,FABP5,KRT1,KRT10,KRT14,KRT27,KRT6B,MARCKS,METAP1,MIF,NDRG1,PCYT1A,RPL14,RPS19,SARS1,SLC2A1,TARS1,YBX3
	EFNA4	kinase	EFNA4	1	Activated	2.449	5.54E-09	0.0001	FABP5,KRT1,KRT10,KRT16,KRT75,PKP1
	EFNA3	kinase	EFNA3	1	Activated	2.449	5.54E-09	0.0001	FABP5,KRT1,KRT10,KRT16,KRT75,PKP1
	EFNA5	kinase	EFNA5	1	Activated	2.449	1.45E-08	0.0001	FABP5,KRT1,KRT10,KRT16,KRT75,PKP1
	EFNA2	kinase	EFNA2	1	Activated	2.449	2.09E-08	0.0001	FABP5,KRT1,KRT10,KRT16,KRT75,PKP1
	MNK1/2	group	MKNK1,MNK1/2,MYC	2	Activated	3.638	6.23E-08	0.0012	ACOT7,CCT3,CD44,DSP,EEF1D,EIF4G1,FABP5,KRT14,KRT27,KRT6B,METAP1,MIF,NDRG1,RPL14,RPS19,SLC2A1,YBX3,YWHAG
	F8	other	F10,F8,IgG	2	Activated	3.000	3.09E-07	0.0002	DSG3,DSP,FABP5,KRT10,KRT16,KRT31,MIF,TACSTD2,TRIM29
	KMT2D	transcription regulator	KMT2D	1	Inhibited	-2.828	3.60E-07	0.0001	DSC2,DSC3,DSG3,KRT10,KRT16,KRT23,KRT6A,KRT6C
	IgG	complex	IgG	1	Inhibited	-2.828	4.34E-07	0.0002	DSG3,DSP,FABP5,KRT10,KRT16,KRT31,TACSTD2,TRIM29
	DSP	other	DSP	1	Activated	2.000	4.36E-07	0.0001	DSC2,DSC3,JUP,PKP1
	TP63	transcription regulator	TP63	1		1.897	6.25E-07	0.0002	KRT1,KRT10,KRT14,KRT31,KRT6A,KRT6B,PKP1,SERPINB5,SFN,TRIM29
	methylprednisolone	chemical drug	methylprednisolone	1	Activated	2.714	1.72E-06	0.0008	DSC2,DSG3,FABP5,JUP,KRT10,LAD1,PCYT1A,PKP1,TACSTD2,TRIM29,YBX3
	EFNA1	other	EFNA1	1	Activated	2.236	1.91E-06	0.0001	FABP5,KRT1,KRT10,KRT75,PKP1
	SFN	other	SFN	1	Activated	2.000	1.93E-06	0.0001	DSC2,DSP,JUP,PKP3
	CDK4/6	group	CDK4/6	1	Inhibited	-2.236	4.06E-06	0.0002	EEF1B2,KRT1,KRT10,MARCKS,PCYT1A
WM27	LARP1	translation regulator	LARP1	1	Inhibited	-2.236	2.94E-08	1.00E-04	EIF3F,RPL22,RPL26,RPS21,RPS25
	chrysotile asbestos	chemical toxicant	AKT1,chrysotile asbestos,CREB1,EIF2AK3,ERK,ESR1,IGF1R,MKNK1,MMP12,MMP2,MMP3,MMP9,MYC,MYCN,P38 MAPK,PGR,RB1,TGFB1,TNF,TP53,VEGFA	3	Activated	2.065	1.08E-07	5.00E-04	ATP5F1D,CALML3,FKBP1A,HSPA2,MYL6B,NACA,PAFAH1B2,PFKP,RAB5A,RAB6A,RPL22,RPL26,RPLP1,RPS21,RPS25,SSR4,TMEM109,TRA2B,UBE2L3
	MYCN	transcription regulator	MYCN	1		1.633	3.06E-07	1.00E-04	NACA,RPL22,RPL26,RPLP1,RPS25,TMEM109
	PPM1B	phosphatase	AKT1,CDK2,CDK6,CREB1,CSF2,DYRK1A,EIF2AK3,EZH2,HSF1,IGF1R,IKBKB,MAPK3,MKNK1,MYC,NFkB (complex),NLRP3,P38 MAPK,PGR,PPM1B,TBK1,TP53,XBP1	3		-1.698	4.01E-06	1.70E-03	AHSA1,ATP5F1D,FKBP1A,HSPA2,NACA,PAFAH1B2,PFKP,RAB5A,RAB6A,RPL26,RPLP1,RPS21,RPS25,SSR4,TRA2B,UBE2L3,UBE2N
	Cdk	group	ACLY,AKT1,CDC20,Cdk,CDK1,CDK2,CDK3,CDK5,CDK6,CDK7,CDK9,CDKN2A,CHEK1,ESR1,EZH2,HSF1,KRAS,MASTL,MYC,NFAT5,NR3C2,PGR,PP1 protein complex group,RB1,TP53	3	Activated	2.000	4.17E-06	2.10E-03	AHSA1,ATP5F1D,FKBP1A,HSPA2,MYL6B,PAFAH1B2,RAB6A,RPL22,RPL26,RPLP1,RPS21,RPS25,TMEM109,TRA2B,TUBB6,UBE2L3
	MLXIPL	transcription regulator	MLXIPL	1	Activated	2.000	7.72E-06	1.00E-04	RPL22,RPL26,RPS21,RPS25
	thymoquinone	chemical drug	AKT1,CASP8,CASP9,CAT,CSF2,HSF1,IL3,IL33,IL5,INSR,MAPK1,MYC,NLRP3,P38 MAPK,Pro-inflammatory Cytokine,RB1,STAT3,TGFB1,thymoquinone	3	Inhibited	-2.138	3.20E-05	3.50E-03	AHSA1,ATP5F1D,CALML3,MYL6B,NACA,RAB5A,RPL22,RPL26,RPLP1,RPS21,TRA2B,TUBB6,UBE2L3,UBE2N
	ZEB	group	AKT1,MYC,P38 MAPK,RB1,TP53,ZEB,ZEB1,ZEB2	3	Activated	2.714	6.30E-05	3.50E-03	FKBP1A,MYL6B,PAFAH1B2,PFKP,RAB5A,RPL22,RPL26,RPLP1,RPS21,RPS25,UBE2L3
	Mir200	group	AKT1,mir-8,Mir200,MYC,P38 MAPK,RB1,TP53,ZEB1,ZEB2	3	Inhibited	-2.714	6.48E-05	3.90E-03	FKBP1A,MYL6B,PAFAH1B2,PFKP,RAB5A,RPL22,RPL26,RPLP1,RPS21,RPS25,UBE2L3
	BIRC5	other	BIRC5,MYC,RB1,TP53,VEGFA	2		1.897	1.70E-04	4.60E-03	FKBP1A,MYL6B,PAFAH1B2,PFKP,RAB5A,RPL22,RPL26,RPLP1,RPS21,RPS25
	RICTOR	other	RICTOR	1	Inhibited	-2.000	2.34E-04	4.00E-03	ATP5F1D,RPL22,RPL26,RPS21
	5-fluorouracil	chemical drug	5-fluorouracil	1	Inhibited	-2.000	3.22E-04	1.90E-03	AHSA1,FKBP1A,NACA,SSR4
	FABP2	transporter	AKT1,CPT1A,FABP2,HMGCR,LIPE,MYC,P38 MAPK	3		1.890	5.54E-04	3.70E-03	RAB5A,RPL22,RPL26,RPLP1,RPS21,RPS25,UBE2L3
	1-octanol	chemical - endogenous mammalian	1-octanol,CSF2,GATA4,HMGCR,MYC,Nfat (family),NFAT5	3	Inhibited	-2.828	5.80E-04	2.70E-03	HSPA2,NACA,PFKP,RPL22,RPL26,RPLP1,RPS21,RPS25
WM28	LARP1	translation regulator	LARP1	1	Inhibited	-3.873	3.59E-32	0.0001	RACK1,RPL10,RPL13,RPL23A,RPL24,RPL4,RPL7,RPS14,RPS15A,RPS20,RPS28,RPS3A,RPS5,RPS6,RPS8
	MLXIPL	transcription regulator	MLXIPL	1	Activated	3.873	8.54E-30	0.0001	RPL10,RPL13,RPL24,RPL4,RPL7,RPLP0,RPS14,RPS15A,RPS20,RPS26,RPS28,RPS5,RPS6,RPS8,RPSA
	MYCN	transcription regulator	MYCN	1	Activated	3.742	3.27E-22	0.0001	EIF4A1,RPL13,RPL23A,RPL24,RPL4,RPL7,RPLP0,RPS20,RPS26,RPS28,RPS3A,RPS5,RPS6,RPS8
	MYC	transcription regulator	MYC	1	Activated	4.000	3.96E-17	0.0001	EIF4A1,RPL10,RPL13,RPL24,RPL4,RPL7,RPLP0,RPS14,RPS15A,RPS20,RPS26,RPS28,RPS5,RPS6,RPS8,RPSA
	sirolimus	chemical drug	sirolimus	1	Inhibited	-3.464	2.96E-14	0.0001	EIF4A1,RACK1,RPL10,RPLP0,RPS14,RPS20,RPS26,RPS3A,RPS5,RPS6,RPS8,RPSA
	RICTOR	other	RICTOR	1	Inhibited	-3.000	4.10E-12	0.0001	RPL10,RPL4,RPL7,RPLP0,RPS26,RPS5,RPS6,RPS8,RPSA
	ST1926	chemical drug	ST1926	1	Inhibited	-2.449	1.20E-08	0.0001	EIF4A1,EIF4A2,RPL10,RPL13,RPL24,RPS26
	TCR	complex	TCR	1	Activated	2.449	1.86E-07	0.0002	RPL23A,RPL4,RPL7,RPLP0,RPS3A,RPSA
	5-fluorouracil	chemical drug	5-fluorouracil	1		-1.633	4.62E-07	0.0002	EIF4A1,RPL10,RPL24,RPL7,RPS15A,RPS8
	CD 437	chemical drug	CD 437	1	Inhibited	-2.236	1.81E-06	0.0001	EIF4A1,EIF4A2,RPL10,RPL13,RPL24
	CAMK2N2	other	AKT1,CAMK2A,CAMK2N2,CaMKII,CCND1,EGFR,FOS,HSF1,IFNG,JUN,LCK,MAPK1,NFE2L2,NFKBIA,PPARG,TP53	3	Inhibited	-2.138	4.28E-06	0.0031	CCT8,EIF4A1,EIF4A2,RACK1,RPL13,RPL23A,RPL4,RPL7,RPLP0,RPS20,RPS26,RPS5,RPSA,SERBP1
	TFAP2A	transcription regulator	TFAP2A	1		-1.732	3.45E-05	0.0001	RPL7,RPLP0,RPS5
	CDK4/6	group	CDK4/6	1		-1.732	5.19E-05	0.0004	RPL10,RPL13,RPS6
	tanespimycin	chemical drug	tanespimycin	1		-1.732	2.59E-04	0.0011	EIF4A1,RPL24,RPS8
	retaspimycin	chemical drug	Hsp90,HSP90B1,retaspimycin	2		-1.732	4.04E-04	0.0015	RPS14,RPS20,RPS6

Table S2. The top master regulators and participating regulators of the three network modules (WM15, WM16, and WM18) significant to the lung SqCC, together with target molecules in the datasets, which were predicted to be activated or inhibited (|z-value|> 2.0) and up-regulated (1.5 < *z*-value < 2.0) with the significance of network bias-corrected *p*-value < 0.005. Chemical drugs are indicated in red letter.

Module ID	Master Regulator	Molecule Type	*Participating regulators*	Depth	Predicted activation	Activation z-score	*p*-value of overlap	Network bias-corrected *p*-value (<0.005)	Target molecules in Dataset
WM15	NUPR1	transcription regulator	NUPR1	1	Inhibited	-2.449	1.23E-05	0.0003	H2BC3,H3C1,H3C15,H4C1,HNRNPA2B1,HNRNPM
	E2F3	transcription regulator	E2F3	1		1.732	4.15E-04	0.0009	H1-2,H2AW,H2BC11
	gentamicin	chemical drug	gentamicin	1	Activated	2.000	4.44E-04	0.0005	H2AX,H3-3A/H3-3B,HNRNPU,PTBP1
	IL12R	group	AKT1,CDK1,CHEK2,IFNG,IKBKB,IL12R,JAK,JUN,MAP2K6,SNAI1,STAT4,TP53	3		-1.508	0.00105	0.0046	H2AC18/H2AC19,H2AC21,H2AX,H2BC18,H2BC3,H2BC5,H3-3A/H3-3B,HNRNPA2B1,HNRNPM,HNRNPU,PEBP1
	AURKA	kinase	AKT1,AURKA,CDK1,MYC	2		1.633	0.00451	0.0083	H2AX,H3-3A/H3-3B,HNRNPA2B1,HNRNPM,HNRNPU,PTBP1
	CAMK2N2	other	AKT1,CAMK2N2,CaMKII,CEBPB,IFNG,JUN,MAPK1,MDM2,MKNK1,SNAI1,TP53	3		1.897	0.0049	0.0204	H2AC18/H2AC19,H2AC21,H2BC18,H2BC3,H2BC5,H4C1,HNRNPM,HNRNPU,PEBP1,PTBP1
	RPL11	other	MDM2,MYC,RPL11,TP53	2		-1.890	0.00534	0.0097	H2AC18/H2AC19,H2AX,H2BC5,H3-3A/H3-3B,HNRNPA2B1,HNRNPU,PTBP1
	amphetamine	chemical drug	Akt,AKT1,amphetamine,CaMKII,ERK1/2,IFNG,MAPK1,MDM2,MKNK1,MYC,PTPRR,SNAI1,TP53	3	Inhibited	-2.530	0.00597	0.0148	H2AC18/H2AC19,H2AC21,H2AW,H2BC18,H2BC3,H2BC5,H3-3A/H3-3B,HNRNPM,PEBP1,PTBP1
	RUNX1T1	transcription regulator	CEBPB,JUN,RUNX1T1	2	Inhibited	-2.000	0.00702	0.0065	H2AX,H4C1,HNRNPA2B1,HNRNPU
	p70 S6k	group	AKT1,CDKN2A,MYC,p70 S6k	2		1.633	0.00744	0.015	H2AX,H3-3A/H3-3B,HNRNPA2B1,HNRNPM,HNRNPU,PTBP1
	IL4	cytokine	AKT1,EGFR,IFNG,IKBKB,IL4,JUN,MAP2K1/2,SFPQ,STAT4,TP53	2		1.897	0.00746	0.0284	H2AC18/H2AC19,H2AC21,H2AX,H2BC18,H2BC3,H2BC5,H3-3A/H3-3B,HNRNPA2B1,HNRNPM,HNRNPU
	PIM2	kinase	ATM,CDKN1A,MDM2,MYC,PIM2	2		1.633	0.0078	0.0138	H2AC18/H2AC19,H2AX,H3-3A/H3-3B,HNRNPA2B1,HNRNPU,PTBP1
	PSMD14	peptidase	26s Proteasome,JUN,PSMD14	2		-1.732	0.0111	0.0048	H2AX,HNRNPA2B1,HNRNPU
	sorafenib analog BB1	chemical - kinase inhibitor	FLT1,NTRK1,RAF1,sorafenib analog BB1	2		-1.732	0.0157	0.0115	H2AX,HNRNPA2B1,PTBP1
	FOXP1	transcription regulator	FOXP1,IFNG,JUN	2	Activated	2.236	0.0176	0.0186	H2AC21,H2BC18,H2BC3,HNRNPA2B1,HNRNPU
	TCL1A	transcription regulator	AKT1,JUN,TCL1A	2		1.732	0.024	0.0201	HNRNPA2B1,HNRNPM,HNRNPU
WM16	IL21	cytokine	Akt,AKT1,CD247,CLOCK,ERK1/2,ESR1,EZH2,FOS,HRAS,IL21,JAK,JAK3,MAP2K3,Mapk,MAPK9,MYC,NFE2L2,Shc,SPARC,STAT3,STAT4,STAT5A,SYK,Vegf	3	Activated	2.840	2.07E-05	0.0004	ANP32A,ARF1,ARL1,COPB2,COPG1,GLG1,KTN1,NIPSNAP2,RBM25,RPL27,RPS17,SCAMP2,SF3B4,TMED2,TMX1
	IFNGR	complex	Calmodulin,EGFR,ESR1,GSK3B,HRAS,Ifn,IFNGR,JAK,JAK2,KAT5,MYC,NFE2L2,PTEN,STAT1,STAT4,VHL	3	Activated	3.357	4.66E-06	0.0007	ANP32A,ARF1,COPB2,COPG1,DYNC1LI2,GLG1,GTF2I,KTN1,MAVS,NSF,PSMC4,RBM25,RPL27,RPS17,TMED2
	N-(3-(aminomethyl) benzyl)acetamidine	chemical reagent	CASP3,CD247,EGFR,FOXO1,HRAS,ITGB2,MAPK9,MYC,N-(3-(aminomethyl) benzyl)acetamidine,NOS1,NOS2,NOS3,PTEN,STAT4,ZAP70	3	Inhibited	-2.714	1.05E-04	0.0011	ARF1,COPB2,COPG1,DYNC1LI2,GTF2I,MAVS,NSF,RPL27,RPS17,SF3B4,SNRNP70
	OGA	enzyme	26s Proteasome,CTNNB1,EGFR,ESR1,EZH2,GSK3B,JUN,NFE2L2,OGA,PPARG,PTEN,tamoxifen,VHL	3	Inhibited	-3.051	3.49E-05	0.0013	ANP32A,ARF1,COPG1,DYNC1LI2,GTF2I,KTN1,MAVS,NSF,PSMC4,RBM25,SCAMP2,SNRNP70,TMED2
	CXCL12	cytokine	CD247,CXCL12,EGFR,ESR1,ESR2,FOS,FOXO1,FOXO3,JUN,KRAS,ZAP70	2	Activated	2.714	4.85E-04	0.0021	ANP32A,ARF1,DYNC1LI2,GTF2I,KTN1,MAVS,NSF,PSMC4,RBM25,RPS17,SNRNP70
	STAT	group	AKT1,CLOCK,ERK,ESR1,ESR2,FOS,FOXO3,MAP2K3,MYC,MYCN,PTEN,SPARC,STAT3,STAT4,STAT5A	3	Activated	3.051	9.46E-05	0.0025	ANP32A,ARF1,ARL1,COPG1,GLG1,GTF2I,KTN1,NIPSNAP2,PSMC4,RBM25,RPL27,RPS17,TMX1
	TIPRL	other	EGFR,FOXO3,GSK3B,IKBKB,KAT5,MAP2K3,MAP3K7,MAPK9,MYC,NFE2L2,PPP2R1A,PTEN,SREBF2,TAB1,TIPRL,VHL	3	Inhibited	-2.714	9.93E-05	0.0028	ARF1,ARL1,DYNC1LI2,GTF2I,MAVS,NSF,PSMC4,RPL27,RPS17,SF3B4,TMED2
	N-methyl-3,4-methylenedioxyamphetamine	chemical drug	26s Proteasome,CYP2D6,ERK1/2,ESR1,FOS,Hsp27,JUN,MAP2K3,MYC,N-methyl-3,4-methylenedioxyamphetamine,NFE2L2,NFkB (complex),PCBP2,tamoxifen	3	Inhibited	-2.887	8.32E-05	0.0032	ANP32A,ARF1,ARL1,DYNC1LI2,KTN1,MAVS,PSMC4,RBM25,RPL27,RPS17,SNRNP70,TMED2
	IFN type 1	group	EGFR,ESR1,ESR2,FOS,Ifn,IFN type 1,MYC,PPARG,STAT1,STAT3,STAT4,STAT5A,STAT5B	3	Activated	3.464	0.000235	0.0041	ANP32A,ARF1,COPG1,DYNC1LI2,GLG1,KTN1,MAVS,NSF,PSMC4,RBM25,RPL27,RPS17
	LOC105377329	other	GSK3B,JUN,KAT5,LOC105377329,MYC,NFE2L2,NFkB (complex),PPARG,PTEN,STAT4,VHL	3	Inhibited	-3.317	0.000421	0.0078	ARF1,COPG1,DYNC1LI2,GTF2I,MAVS,NSF,PSMC4,RPL27,RPS17,SNRNP70,TMED2
	MEF2D-NFAT2-p300	complex	EP300,ESR1,FOXO1,JUN,MEF2D-NFAT2-p300,NFATC1,NFE2L2,PPARG,XBP1	3	Activated	3.162	0.00231	0.01	ANP32A,ARF1,COPB2,DYNC1LI2,KTN1,MAVS,NSF,RBM25,SNRNP70,TMED2
WM18	lenalidomide	chemical drug	lenalidomide	1	Activated	2.000	0.0000289	0.0003	HLA-DRB1,HLA-DRB3,HLA-DRB4,SEPTIN2,UTRN
	NR1H3	ligand-dependent nuclear receptor	AKT1,NR1H3,PPARA	2	Inhibited	-2.000	0.000169	0.0014	ABCD3,ASL,COL8A1,FGA,GSTK1,HLA-DRB4,HSD17B4
	apomorphine	chemical drug	AKT1,apomorphine,FOS	2	Inhibited	-2.000	0.000586	0.0017	ASL,COL8A1,FGA,HLA-DRB4,LTBP2,SEC23B
	oxymetazoline	chemical drug	ADRA1A,ADRA1B,ADRA1D,ADRA2A,ADRA2B,ADRA2C,Akt,FOS,HTR1B,MAPK1,oxymetazoline,STAT3,TGM2,voltage-gated calcium channel	3	Activated	2.236	0.0000498	0.0031	ASL,FGA,GSTK1,ITPR3,LTBP2,PDLIM3,POR,RAB27A,SEC23B,UTRN
	MAPK8IP1	other	AKT1,APP,MAPK8,MAPK8IP1,NFATC1	2	Activated	2.000	0.000783	0.0072	ABCD3,COL8A1,DPYSL2,HLA-DRB4,ITPR3,MVP,UTRN
	GABA receptor	complex	Akt,AKT1,calpain,FOS,GABA receptor,GABBR1,IL1A,voltage-gated calcium channel	3	Activated	2.236	0.00032	0.0097	ASL,COL8A1,FGA,GSTK1,HLA-DRB4,ITPR3,LTBP2,SEC23B,UTRN
	complement receptor	complex	Akt,AKT1,BCAR1,C3AR1,C5AR1,Ca2+,CEBPB,complement receptor,ERBB2,Focal adhesion kinase,INSR,ITGAM,ITGB2,MAPK1,NFkB (complex),NFKBIA,PLC,PTK,RET,STAT3,TP53	3		1.897	0.0000205	0.0156	ABCD3,ASL,COL8A1,DPYSL2,FGA,GSTK1,HLA-DRB4,HSD17B4,ITPR3,LPCAT1,LTBP2,OGDH,PDLIM3,RAB27A,SEC23B,UTRN
	NCOA3	transcription regulator	Akt,ERBB2,ESR1,genistein,NCOA3,PGR,T3-TR-RXR,tamoxifen,THRB	2		1.897	0.000131	0.0195	ABCD3,COL8A1,DPYSL2,FGA,GSTK1,LPCAT1,LTBP2,MVP,OGDH,POR,RAB27A,RRBP1,UTRN
	ergotamine	chemical drug	ADRA1A,ADRA1B,ADRA1D,ADRA2A,ADRA2B,ADRA2C,Akt,cyclic AMP,CYP3A4,ergotamine,FOS,HTR1A,HTR1B,MAPK1,STAT3,TGM2,voltage-gated calcium channel	3	Activated	2.236	0.000308	0.02	ASL,FGA,GSTK1,ITPR3,LTBP2,PDLIM3,POR,RAB27A,SEC23B,UTRN
	pictilisib	chemical drug	Akt,AKT1,HIF1A,pictilisib,STAT3,Vegf	2	Activated	2.000	0.00119	0.0221	COL8A1,ERGIC1,FGA,GSTK1,HLA-DRB4,ITPR3,RAB27A,UTRN
	adenosine	chemical - endogenous mammalian	adenosine,Akt,AKT1,CFTR,NFkB (complex),PLAT,STAT3,voltage-gated calcium channel	2	Activated	2.449	0.000424	0.025	ABCD3,COL8A1,FGA,HLA-DRB4,HSD17B4,ITPR3,MVP,POR,RAB27A,UTRN
	metribolone	chemical reagent	metribolone	1	Activated	2.000	0.00406	0.0295	GALE,HSD17B4,POR,RAB27A
	Trolox C	chemical drug	Akt,AKT1,MAPK8,Trolox C	2	Inhibited	-2.000	0.00962	0.0328	COL8A1,GSTK1,HLA-DRB4,MVP,UTRN
	ICMT	enzyme	AKT1,APP,ICMT,KRAS	2	Inhibited	-2.000	0.00488	0.0351	ABCD3,COL8A1,DPYSL2,HLA-DRB1,HLA-DRB4,ITPR3
	KIN001-043	chemical - kinase inhibitor	Akt,AKT1,KIN001-043,STAT3	2	Activated	2.000	0.00568	0.0387	COL8A1,FGA,GSTK1,HLA-DRB4,RAB27A,UTRN
	modafinil	chemical drug	acetaminophen,Akt,cyclophosphamide,CYP2B6,CYP2C9,CYP3A4,CYP3A5,MAPK8,modafinil,SLC6A3,STAT3	3	Inhibited	-2.000	0.00394	0.0389	ASL,FGA,GSTK1,HSD17B4,MVP,RAB27A,UTRN

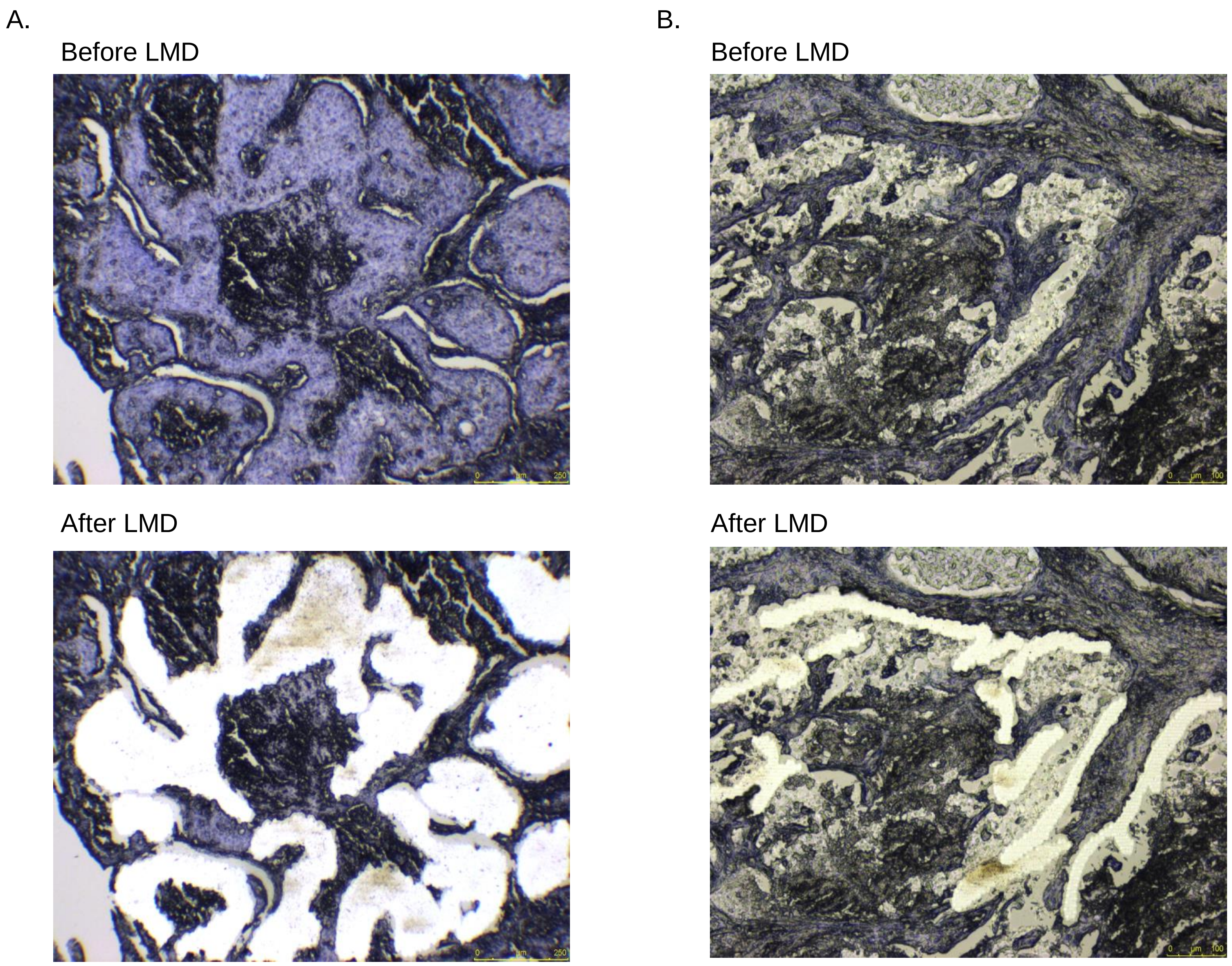

Figure S1. Representative images of tumour tissues from lung SqCC and PPA stained with hematoxylin using standard histological methods prior to dissection. A) SqCC (sample SqCC_T62), B) PPA (sample PPA_T12). Scale bars in yellow color are indicated at the bottom right.

Module ID	Module color	#proteins	*Correlation* SqCC	PPA	*p*-value	BH-corrected *q*-value
WM1	black	53	-0.243	0.243	0.3830	0.5223
WM2	grey60	31	-0.070	0.070	0.8046	0.8046
WM3	steelblue	16	-0.477	0.477	0.0723	0.1807
WM4	brown	77	-0.243	0.243	0.3819	0.5456
WM5	salmon	40	-0.185	0.185	0.5085	0.5868
WM6	pink	51	-0.206	0.206	0.4611	0.5534
WM7	royalblue	29	-0.114	0.114	0.6856	0.7345
WM8	darkgrey	24	-0.180	0.180	0.5211	0.5790
WM9	tan	41	-0.111	0.111	0.6931	0.7170
WM10	cyan	37	-0.479	0.479	0.0710	0.1938
WM11	magenta	49	-0.646	0.646	0.0093	0.0348
WM12	turquoise	83	-0.210	0.210	0.4516	0.5645
WM13	white	21	-0.695	0.695	0.0040	0.0201
WM14	lightgreen	31	-0.225	0.225	0.4191	0.5466
WM15	darkgreen	26	-0.764	0.764	0.0009	0.0055
WM16	darkorange	22	-0.923	0.923	9.662E-07	2.898E-05
WM17	darkred	27	-0.622	0.622	0.0133	0.0445
WM18	midnightblue	37	-0.689	0.689	0.0045	0.0194
WM19	lightyellow	30	0.312	-0.312	0.2579	0.5159
WM20	purple	48	0.367	-0.367	0.1789	0.3834
WM21	yellow	66	0.309	-0.309	0.2626	0.4635
WM22	red	59	0.304	-0.304	0.2701	0.4502
WM23	green	61	0.297	-0.297	0.2817	0.4448
WM24	skyblue	17	0.311	-0.311	0.2591	0.4858
WM25	lightcyan	32	0.288	-0.288	0.2973	0.4460
WM26	blue	80	0.851	-0.851	5.726E-05	0.0004
WM27	darkturquoise	26	0.900	-0.900	4.892E-06	7.339E-05
WM28	orange	24	0.884	-0.884	1.230E-05	0.0001
WM29	greenyellow	44	0.501	-0.501	0.0572	0.1715
WM30	saddlebrown	17	0.391	-0.391	0.1491	0.3441

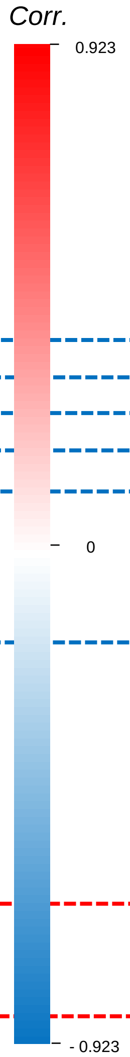

Figure S2. Relationship between module eigen-proteins and the clinical traits of subtypes SqCC and PPA.

Each row in the embedded table represents weighted gene co-expression network analysis results for each module. The first and second columns in the table represent the module identification and colour name of the module, respectively. The third column represents the number of proteins in each module. The table is colour-coded by the correlation coefficient according to the colour legend on the right side of the fifth column, in which the intensity and direction of the correlations are indicated (red, positive correlation; blue, negative correlation). The *p*-values of the correlation coefficients and *q*-values by multiple testing correction using the Benjamini–Hochberg method are presented. The sixth and seventh columns with significant *p*- and *q*-values (< 0.05) are highlighted in bright red background.

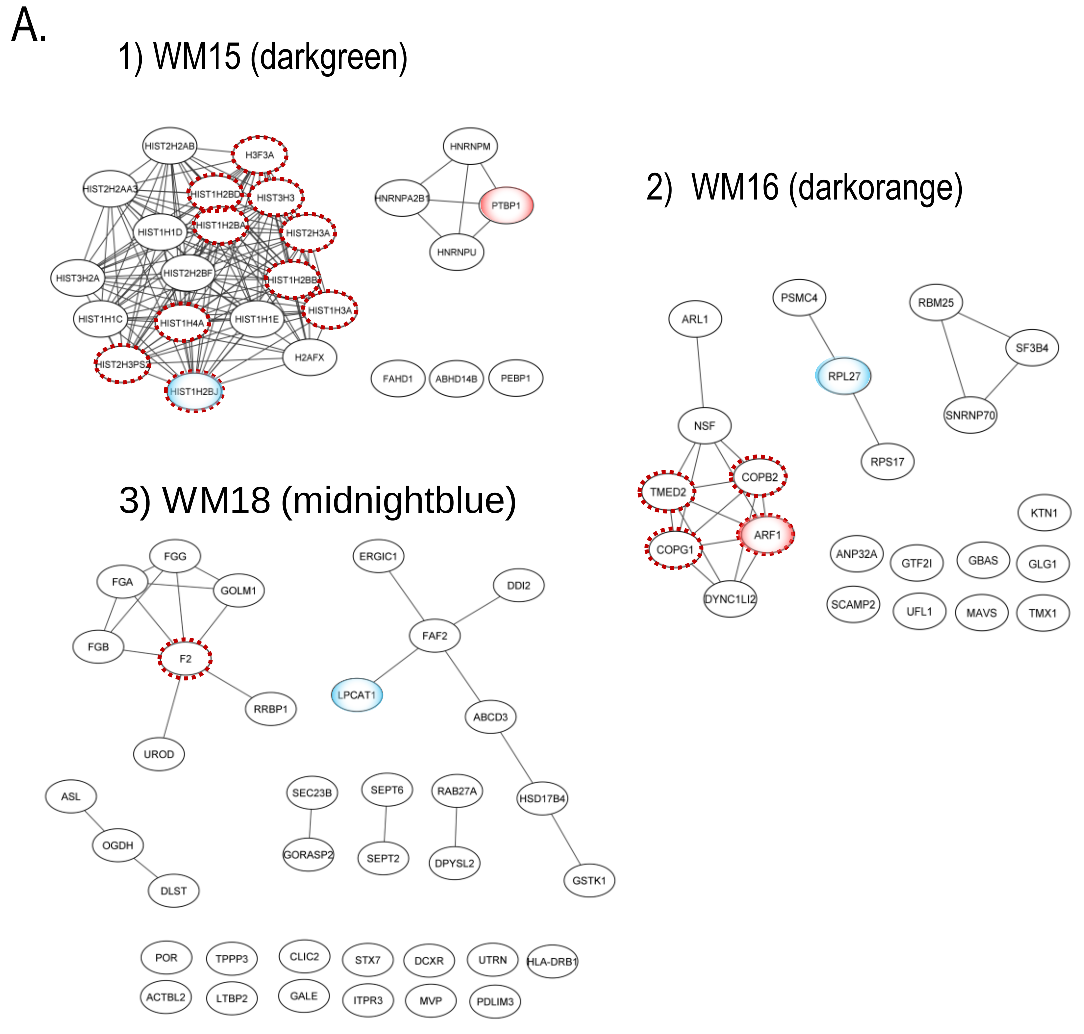

B.

Module ID	No.	# Term ID[a)]	Term description	Strength[b)]	False discovery rate (FDR)	BH-corrected *q*-value[c)]
WM15 (darkgreen)	1	CL:11119	nucleosome	2.1	8.97E-29	1.10E-26
	2	CL:11122	nucleosome	2.13	1.08E-25	6.64E-24
	3	CL:11125	nucleosome	2.13	5.89E-24	2.41E-22
	**4**	**GO:0006333**	**chromatin assembly or disassembly**	**1.83**	**3.79E-22**	**1.17E-20**
	5	GO:0034728	nucleosome organization	1.85	3.79E-22	9.32E-21
	6	GO:0006334	nucleosome assembly	1.9	1.33E-21	2.73E-20
	7	GO:0071103	DNA conformation change	1.66	3.06E-20	5.38E-19
	8	GO:0006325	chromatin organization	1.31	2.42E-19	3.72E-18
	9	GO:0051276	chromosome organization	1.17	3.03E-18	4.14E-17
	**10**	**CL:11226**	**Histone H2A/H2B/H3**	**2.47**	**6.27E-17**	**7.71E-16**
	**11**	**HSA-2559583**	**Cellular Senescence**	**1.77**	**8.09E-17**	**9.05E-16**
	**12**	**HSA-2559586**	**DNA Damage/Telomere Stress Induced Senescence**	**2.11**	**8.09E-17**	**8.29E-16**
	13	HSA-2299718	Condensation of Prophase Chromosomes	2.22	2.2E-16	2.08E-15
	14	HSA-912446	Meiotic recombination	2.11	1.48E-15	1.30E-14
	15	HSA-201722	Formation of the beta-catenin:TCF transactivating complex	2.07	2.43E-15	1.99E-14
	**16**	**HSA-3214815**	**HDACs deacetylate histones**	**2.07**	**2.43E-15**	**1.87E-14**
	**17**	**HSA-73728**	**RNA Polymerase I Promoter Opening**	**2.29**	**2.76E-15**	**2.00E-14**
	**18**	**HSA-5334118**	**DNA methylation**	**2.27**	**3.7E-15**	**2.53E-14**
	19	HSA-5625886	Activated PKN1 stimulates transcription of AR (androgen receptor) regulated genes KLK2 and KLK3	2.24	4.93E-15	3.19E-14
	**20**	**HSA-427359**	**SIRT1 negatively regulates rRNA expression**	**2.23**	**5.4E-15**	**3.32E-14**
WM16 (darkorange)	**1**	**HSA-6807878**	**COPI-mediated anterograde transport**	**1.73**	**3.40E-07**	**1.12E-05**
	2	HSA-6811442	Intra-Golgi and retrograde Golgi-to-ER traffic	1.5	3.40E-07	5.61E-06
	**3**	**HSA-8856688**	**Golgi-to-ER retrograde transport**	**1.62**	**5.18E-07**	**5.70E-06**
	**4**	**HSA-6811434**	**COPI-dependent Golgi-to-ER retrograde traffic**	**1.67**	**3.21E-06**	**2.65E-05**
	5	GO:0048193	Golgi vesicle transport	1.28	4.28E-05	2.82E-04
	6	HSA-392499	Metabolism of proteins	0.7	7.18E-05	3.95E-04
	7	HSA-597592	Post-translational protein modification	0.77	1.90E-04	8.96E-04
	**8**	**CL:18556**	**COPI-coated vesicle membrane, and COPI coating of Golgi vesicle**	**2.06**	**3.40E-04**	**1.40E-03**
	9	GO:0006888	endoplasmic reticulum to Golgi vesicle-mediated transport	1.41	6.00E-04	2.20E-03
	10	GO:0006890	retrograde vesicle-mediated transport, Golgi to endoplasmic reticulum	1.64	6.30E-04	2.08E-03
	11	HSA-8953854	Metabolism of RNA	0.91	1.30E-03	3.90E-03
	12	GO:0046907	intracellular transport	0.76	2.10E-03	5.78E-03
	**13**	**CL:18558**	**COPI vesicle coat, and endoplasmic reticulum-Golgi intermediate compartment organization**	**2.1**	**3.10E-03**	**7.87E-03**
	**14**	**CL:19049**	**Sec7 domain, and Small GTPase superfamily, ARF/SAR type**	**1.95**	**4.20E-03**	**9.90E-03**
	15	GO:0008104	protein localization	0.61	1.18E-02	2.60E-02
	16	GO:0015031	protein transport	0.71	1.18E-02	2.43E-02
	**17**	**GO:0048024**	**regulation of mRNA splicing, via spliceosome**	**1.54**	**1.18E-02**	**2.29E-02**
	18	GO:1903311	regulation of mRNA metabolic process	1.17	1.18E-02	2.16E-02
	19	HSA-5663205	Infectious disease	0.99	1.24E-02	2.15E-02
	**20**	**CL:16004**	**spliceosomal snRNP complex**	**1.66**	**1.29E-02**	**2.13E-02**
WM18 (midnightblue)	1	HSA-140875	Common Pathway of Fibrin Clot Formation	1.98	2.68E-05	3.43E-03
	2	HSA-76009	Platelet Aggregation (Plug Formation)	1.76	8.90E-05	5.70E-03
	3	CL:9934	Fibrinogen alpha/beta chain family, and Peptidase S1A, plasmin	2.42	8.92E-05	3.81E-03
	**4**	**HSA-354194**	**GRB2:SOS provides linkage to MAPK signaling for Integrins**	**2.09**	**0.00015**	**4.80E-03**
	**5**	**HSA-372708**	**p130Cas linkage to MAPK signaling for integrins**	**2.05**	**0.00015**	**3.84E-03**
	6	GO:0042730	fibrinolysis	2	0.00017	3.63E-03
	**7**	**GO:0072378**	**blood coagulation, fibrin clot formation**	**1.89**	**0.00021**	**3.84E-03**
	8	HSA-5686938	Regulation of TLR by endogenous ligand	1.92	0.00027	4.32E-03
	9	CL:9922	Formation of Fibrin Clot (Clotting Cascade), and platelet alpha granule lumen	1.56	0.00032	4.55E-03
	**10**	**CL:9723**	**Regulation of Insulin-like Growth Factor (IGF) transport and uptake by Insulin-like Growth Factor Binding Proteins (IGFBPs), and Formation of Fibrin Clot (Clotting Cascade)**	**1.21**	**0.00044**	**5.63E-03**
	11	HSA-216083	Integrin cell surface interactions	1.41	0.00048	5.59E-03
	12	HSA-9006921	Integrin signaling	1.8	0.00048	5.12E-03
	13	HSA-6802948	Signaling by high-kinase activity BRAF mutants	1.66	0.00086	8.47E-03
	14	GO:0010811	positive regulation of cell-substrate adhesion	1.38	0.00097	8.87E-03
	15	GO:0030168	platelet activation	1.34	0.00097	8.28E-03
	16	GO:0031639	plasminogen activation	2.16	0.00097	7.76E-03
	17	GO:0034116	positive regulation of heterotypic cell-cell adhesion	2.09	0.00097	7.30E-03
	18	HSA-168249	Innate Immune System	0.67	0.0011	7.82E-03
	19	HSA-381426	Regulation of Insulin-like Growth Factor (IGF) transport and uptake by Insulin-like Growth Factor Binding Proteins (IGFBPs)	1.24	0.0011	7.41E-03
	**20**	**HSA-5674135**	**MAP2K and MAPK activation**	**1.61**	**0.0011**	**7.04E-03**

a) # of term ID: GO, Biological process; HAS, Reactome pathway; CL, STRING local network cluster. b) Strength (Log10(observed / expected), a measure of the enrichment effect, that is the ratio between i) the number of proteins in a network that are annotated with a term and ii) the number of proteins expected to be annotated with this term in a random network of the same size. c) Multiple testing correction using the Benjamini-Hochberg method.

Figure S3. Data-driven protein co-expression networks and pathway enrichment results obtained for the lung PPA trait. A. The co-expression networks of respective modules: 1) WM15, 2) WM16, and 3) WM18 modules. Circle nodes in blue gradation and dotted red represent eigen-proteins and/or hub proteins for the respective module, respectively, and circle nodes in red gradation also indicate key proteins in the network modules. B. Among pathways enriched for the protein core networks obtained for biological process (GO) and Reactome pathways, top 20 pathways are presented in the order of significance by q-value. Dashed circles denote subnetworks and protein groups.

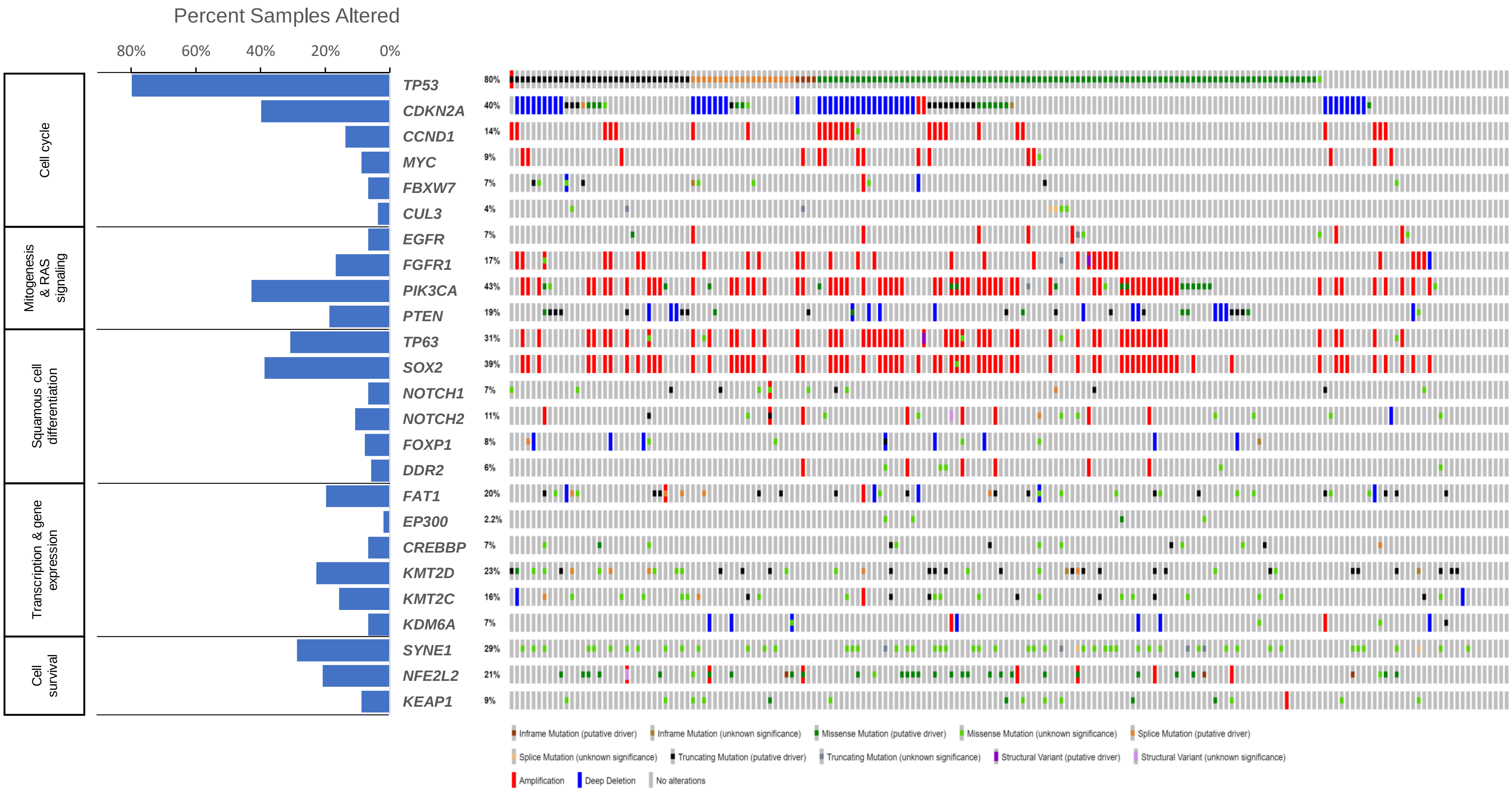

Figure S4. Genomic alteration landscape of early-stage SqCCs obtained from TCGA lung squamous cell carcinoma sub-datasets (T1A-T2A: *n* = 184). Percentage of samples with genomic alterations are presented at the left.

Figure S5. Gene expressions in mRNA level and genomic alterations (types of mutations and copy number variations) of early-stage SqCCs obtained from TCGA lung squamous cell carcinoma sub-datasets (T1A-T2A: n = 184). A) *TP53*, b) *CDKN2A*, c) *MYC*, d) *PIK3CA*, e) *PTEN*, and f) *TP63*.

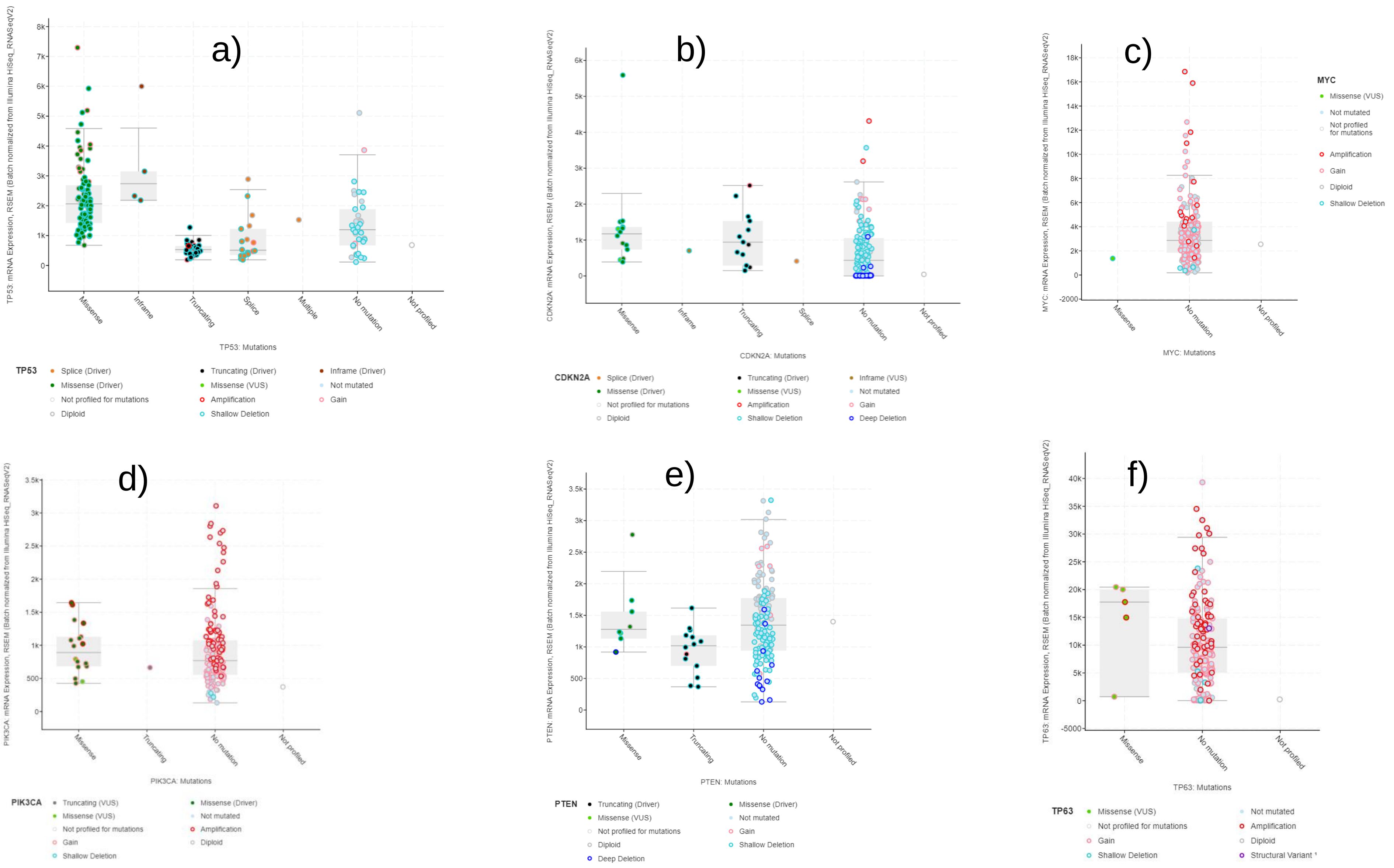

Figure S5. Continued. g) *SOX2*, h) *KMT2D*, and i) *NFE2L2*.

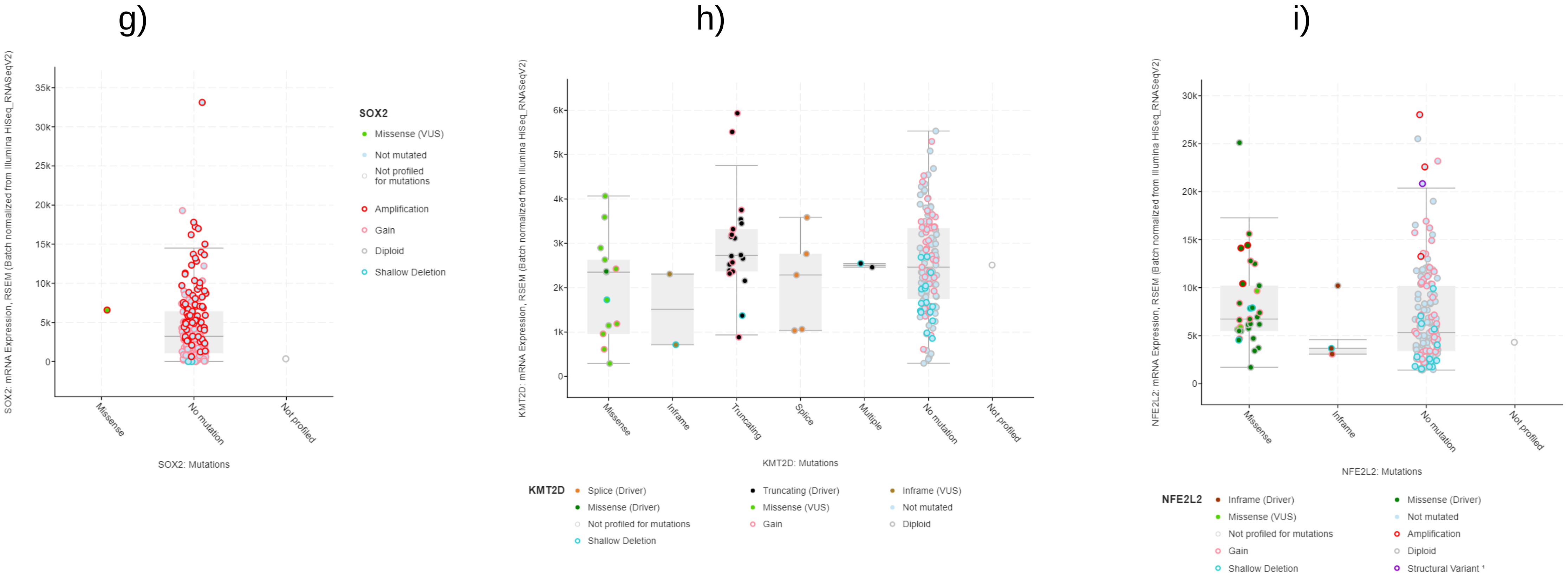

Supplement: Supplementary file 1 — Supplementary Information. [file 41598_2021_99695_MOESM1_ESM.pdf]
